# Interactions of nasal epithelium with macrophages and dendritic cells variously alter urban PM-induced inflammation in healthy, asthma and COPD

**DOI:** 10.1038/s41598-021-92626-w

**Published:** 2021-06-24

**Authors:** Magdalena Paplinska-Goryca, Paulina Misiukiewicz-Stepien, Malgorzata Proboszcz, Patrycja Nejman-Gryz, Katarzyna Gorska, Elwira Zajusz-Zubek, Rafal Krenke

**Affiliations:** 1 Department of Internal Medicine, Pulmonary Diseases and Allergy, Medical University of Warsaw, Banacha 1a, 02-097 Warsaw, Poland; 2 Postgraduate School of Molecular Medicine, Medical University of Warsaw, Warsaw, Poland; 3 Faculty of Energy and Environmental Engineering, Department of Air Protection, Silesian University of Technology, Gliwice, Poland

**Keywords:** Environmental sciences, Medical research, Molecular medicine

## Abstract

Urban particulate matter (UPM) is an important trigger of airway inflammation. The cross-talk between the external and internal matrix in the respiratory tract occurs due to the transepithelial network of macrophages/dendritic cells. This study characterized the immune processes induced by the epithelium after UPM exposure in special regard to interactions with monocyte-derived dendritic cells (moDCs) and monocyte-derived macrophages (moMφs) in obstructive lung diseases. A triple-cell co-culture model (8 controls, 10 asthma, and 8 patients with COPD) utilized nasal epithelial cells, along with moMφs, and moDCs was exposed to UPM for 24 h. The inflammatory response of nasal epithelial cells to UPM stimulation is affected differently by cell–cell interactions in healthy people, asthma or COPD patients of which the interactions with DCs had the strongest impact on the inflammatory reaction of epithelial cells after UPM exposure. The epithelial remodeling and DCs dysfunction might accelerate the inflammation after air pollution exposure in asthma and COPD.

## Introduction

Environmental conditions significantly affect human health. Air pollution may alter the health status on different levels (individual patients and societies) and based on different mechanisms. These include not only a harmful effect but also changes in gene expression as well as metabolic and inflammatory pathways in the respiratory and cardiovascular system. The increased morbidity and mortality caused by air pollution are largely related to exposure to particulate matter (PM) with an aerodynamic diameter from 10 μm (PM_10_) to less than 2.5 μm (PM_2.5_), especially in people with pre-existing obstructive lung diseases^1,2^. Urban particulate matter (UPM) is a mixture of predominating PM_10_ and less amount of PM_2.5_^3^. When inhaled, large PM are mainly deposited in conducting airways where the smallest particles can even reach small peripheral airways, including terminal bronchioles and alveoli. Several factors were identified in PM_2.5_ from rural and urban areas including secondary sulphate, secondary nitrate, biomass burning, gasoline combustion, diesel combustion, dust, industry and winter salt^4^. The air pollutants are significantly associated with an increased risk of asthma exacerbation and hospitalizations^5^. Associations between chronic obstructive pulmonary disease (COPD) and air pollution have also been widely reported^6^. It was shown that chronic exposure to PM_2.5_ resulted in decreased lung function, development of emphysematous lesions and enhancement of airway inflammation in COPD patients^7^.

The mechanisms of the harmful effect of ambient PM on the airways of asthma and COPD patients have not been sufficiently explained. This refers in particular to the interactions between PM and airway epithelial cells that form a layer of direct contact between airway pollution and the human respiratory tract. Due to a variety of inhaled chemical compounds, complex organization and multiple interactions occurring within airway epithelium, the tissue could be affected in many deleterious ways. A Fe-rich PM_10_ fraction from an underground railway station can penetrate the mucus layer and enter primary bronchial epithelial cells (PBEC)^8^. Bronchial epithelial cells exposed to increasing concentrations of urban PM_10_ containing Cu, Ni, Zn and endotoxins show significantly enhanced dose-dependent production of IL-8.

PM_10_ and PM_2.5_ exposure induces oxidative stress and inflammation in the airways. Many animal and in vitro studies reported increased mRNA expression and protein secretion of IL-1α, IL-1β, IL-6, IL-8, TNF-α in epithelial cells after treatment with PM^9–11^. Some research indicated the acceleration of Th2 dependent inflammation in the airways after air pollutant exposure as well as upregulation of TSLP and IL-33 expression^12,13^. Moreover, PM_10_ stimulation is related to epithelial cell dysfunction and remodeling by inducing an increased matrix metalloproteinases (MMPs) activity and downregulation of E-cadherin/b-catenin or claudin in these cells^14,15^. Chronic exposure to air pollution contributes to airway remodeling and mucus hypersecretion^16,17^. The expression of epidermal growth factor receptor (EGFR) ligands was upregulated in human airway epithelial cells exposed to urban PM_2.5_^18^. Additionally, PM_2.5_ had been connected to epithelial-mesenchymal transition (EMT) pathogenesis. The components of PM_2.5_ activated EMT related gene expression (e.g. transforming growth factor β (TGF-β), extracellular signal-regulated protein kinase (ERK), phosphatidylinositol 3-kinase (PI3K), high mobility group box B1 (HMGB1), receptor for advanced glycation end-products (RAGE), aryl hydrocarbon receptor (AHR)) in epithelial cells, which finally leads to changes in the morphology and functions of airway epithelia^19^. All these mechanisms contribute to epithelium dysfunction, which is a dominant factor of obstructive lung disease pathophysiology.

An important component of airway defence is a transepithelial cellular network, which includes macrophages and dendritic cells (DCs). These cells play the role of sentinels against foreign environmental antigens. Macrophages simultaneously encounter inhaled stimuli and regulate the local inflammatory response. Exposure to medium from PM_10_ treated alveolar macrophages (AMs) upregulated the mRNA expression of IL-1β, leukemia inhibitory factor (LIF) and IL-8 in human bronchial epithelial cells (HBEC)^20^. Co-cultivation of AMs with HBEC exposed to PM_10_ increased the production and release of tumor necrosis factor alpha (TNF-α), granulocyte–macrophage colony-stimulating factor (GM-CSF), IL-1β, IL-6, IL-8, LIF and oncostatin M in comparison to control co-cultures.

Understanding how information about UPM signaling is processed and translated into distinct cellular mechanisms remains elusive. In order to better explore cellular responses and interactions in patients with obstructive airway diseases, we undertook a study aimed at the evaluation of (1) the immune response (IL-1β, IL-6, IL-8, MMP7, MMP9, TSLP, IL-33) of airway epithelial cells co-cultured with monocyte-derived dendritic cells (moDCs) and monocyte-derived macrophages (moMφs) to UPM stimulation, (2) the impact of air pollutants on epithelial integrity and remodeling markers expression (TGF-β, EGFR, ST2) in triple co-cultures.

## Material and methods

### Patients’ characteristics

This was a prospective, cross-sectional study, which involved 8 healthy controls, 10 asthma patients, and 8 patients with COPD. In all patients, the diagnosis of asthma or COPD was previously established according to the current recommendations of the Global Initiative for Asthma (GINA) and the Global Initiative for Chronic Obstructive Lung Disease (GOLD)^21,22^. Details on the patients' examination are available in the supplementary file. The control group consisted of smoking and non-smoking volunteers, with normal spirometry. The clinical characteristics of patients and controls recruited for the study are summarized in Table 1. Nasal brushing and peripheral blood samples were obtained from each participant. The study protocol was approved by the Ethics Committee of the Medical University of Warsaw (KB/37/2020) and informed written consent was obtained from all the participants.

**Table 1:** The patients’ characteristics.

	Control n = 8	Asthma n = 10	COPD n = 8	Overall* p* value^a^	Pairwise* p* value*		
					Asthma versus control	COPD versus control	Asthma versus COPD
Age (years)	38.5 (32.5–48)	55 (38–62)	62 (59.5–72.5)	0.005	0.138	0.0002	0.138
Gender (F/M)	6/2	3/10	5/3	0.046			
BMI (kg/m^2^)	22.1 (20.7–24.1)	26.9 (26–27.7)	28 (25.4–30.3)	0.002	0.002	0.0003	0.696
Atopy (n)	3	8	2	0.03			
Smoking exposure (pack-years)	0 (0–0)	0 (0–4)	32.5 (22.5–50)	0.0002	0.277	0.0002	0.0003
FEV_1_ (% predicted)	103 (81–111)	81 (75–94)	61.5 (51.5–76.5)	0.006	0.043	0.004	0.034
FEV_1_/VC (%)	106 (81.8–112)	82 (75–86)	54 (50–68)	0.0003	0.02	0.0006	0.0004
FeNO (ppb)	11.0 (9.3–12.6)	52.3 (31.3–77.6)	17.4 (12.6–26.1)	0.0047	0.03	0.333	0.003
ACT (points)	N.A	20.5 (17–25)	N.A	N.A	N.A	N.A	N.A
ICS treatment (n)	N.A	6	1	N.A	N.A	N.A	N.A
CAT (points)	N.A	N.A	10.5 (8–15)	N.A	N.A	N.A	N.A
mMRC (points)	N.A	N.A	1.5 (1–3)	N.A	N.A	N.A	N.A

Data are presented as median (IQR) or n.

*ACT* Asthma control test, *BMI* body mass index, *FeNO* fractional exhaled nitric oxide FEV_1_ forced expiratory volume at first second, *ICS* inhaled corticosteroids, *mMRC* modified Medical Research Council, *N.A.* not applicable, *VC* vital capacity.

^a^Kruskal Wallis or Chi square test, *Mann–Whitney U test.

### Cell cultures: epithelial cells in an air–liquid interface (ALI) with monocyte-derived dendritic cells (moDCs) or monocyte-derived macrophages (moMφs) culture and triple co-culture

**Figure 1:**
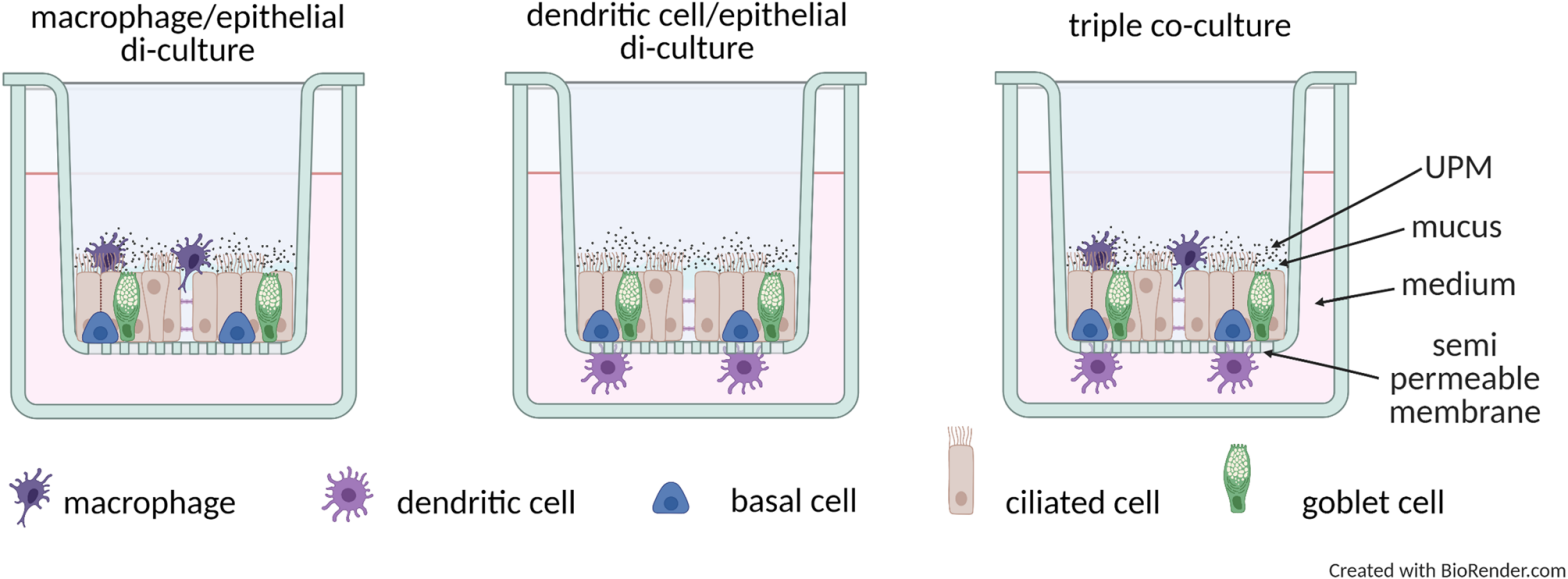
The scheme of di- and triple-co-cultures used in the study.

The study used a triple-cell co-culture model: the nasal epithelial cells cultured in air–liquid interface (ALI) conditions were grown on a microporous membrane in a two-chamber system (Greiner Bio-One, Austria) with monocyte-derived macrophages (moMφs) placed on the top and monocyte-derived dendritic cells (moDCs) placed underneath the epithelial monolayer (Fig. 1). moMφs added on the apical side of the epithelial monolayer were suspended in 35 μl of macrophage medium DXF (Promocell, Germany). moDCs attached to the basal site of inserts with epithelium monolayer were cultured for 24 h in ALI maintenance medium (Stemcell, Canada). Each experiment was performed on triple-co-cultures containing nasal epithelial cells, moMφs as well as moDCs of the same individual. Nasal epithelial cells were obtained by brushing (Cytobrush Plus GT, CooperSurgical, Germany) the inferior surface of the middle turbinate of both nostrils. Macrophages and DCs were specialized from monocytes obtained from a peripheral blood sample. The cells were isolated, cultivated and specialized as previously described^23^ with the protocols of the procedures available in the supplementary file.

### The overall scheme of the study

Epithelial cells were cultured with or without stimulation with 100 µg/ml UPM for 24 h as follows:

1. Epithelial cells,
2. +moDCs (co-culture),
3. +moMφs (co-culture),
4. +moMφs + moDCs (triple co-culture).

The scheme of di- and triple-co-culture models used in the study is illustrated on Fig. 1.

Additionally, a combination with previously 24 h UPM treated moMφs was used in co-cultures of:

1. moMφs (24 h UPM) with unstimulated epithelium,
2. moMφs (24 h UPM) + moDCs with UPM stimulated epithelium.

### Particles preparation

Urban particulate matter was obtained from the Silesian University of Technology. The samples were collected with a low-volume PM sampler type PNS-15 (Atmoservice) 1.5 m above the ground, at a flow rate of 2.3m^3^ h^−1^ in Zabrze, Gliwice or Żory during heating season^24^. PM samples were collected on high-purity quartz (SiO_2_) microfiber filters (QM-A Whatman). The filters were weighed, cut into small pieces, suspended in PBS; the particles were detached from filters by sonication, and autoclaved.

### UPM trace elements analysis

The QM-A filter was mineralized under high pressure and high temperature in a system for microwave mineralization in 8 ml HNO_3_ and 2 ml H_2_O_2_ (Merck, Germany). Elemental concentrations of nine elements (Zn, Fe, Mn, Pb, Cd, Cu, Cr, Ni and Co) were analysed for each sample by atomic absorption spectrometry (Avanta PM, GBC Scientific Equipment Pty Ltd, Melbourne, Australia)^24,25^. The concentrations of heavy metals in UPM are shown in Table 2.

**Table 2:** The characteristic of heavy metals in UPM.

Elements	Concentration
Dust mass (g)	3.65 (3.13–4.08)
V (m^3^)	55.11 (54.60–55.11)
**Zn**	
ng/m^3^	102.89 (97.19–161.94)
µg/g	1467.48 (1063.76–2075.17)
**Fe**	
ng/m^3^	306.47 (232.74–386.93)
µg/g	4557.63 (3479.91–5727.20)
**Mn**	
ng/m^3^	17.02 (13.11–21.30)
µg/g	222.44 (182.32–271.68)
**Pb**	
ng/m^3^	39.55 (34.77–64.25)
µg/g	562.24 (434.74–737.63)
**Cd**	
ng/m^3^	5.96 (4.63–8.10)
µg/g	93.45 (65.42–106.48)
**Cu**	
ng/m^3^	11.70 (9.66–14.15)
µg/g	143.93 (126.53–199.25)
**Cr**	
ng/m^3^	51.58 (40.52–70.65)
µg/g	662.14 (589.04–832.97)
**Ni**	
ng/m^3^	78.88 (57.98–88.90)
µg/g	1032.39 (653.65–1406.09)
**Co**	
ng/m^3^	22.73 (22.68–23.00)
µg/g	342.0 (306.50–400.0)

The results are presented as median and IQR.

### Cell viability

Cell viability was determined by a fluorescence cell counter (Luna, Logos Biosystems, South Korea) using acridine orange/propidium iodide (PI) staining and a flow cytometric using annexin/PI staining (Alexa Fluor 488 annexin V/PI, Thermo Fisher, MA, USA) according to the manufacturer's protocol.

### Measurements of cytokine mRNA expression changes

Quantitative real-time PCR was performed to assess the mRNA expression of IL-1β, IL-6, IL-8, MMP7, MMP9, TSLP, IL-33 and 18 s rRNA in epithelial cells. The mean ΔCT of unstimulated epithelial cells from controls was used as a calibrator for all groups. A detailed protocol of total RNA isolation, cDNA synthesis and real-time PCR is described in the supplementary file and Table S1. The fold changes < 1 were converted and presented as negative values.

### Protein concentration measurements

The levels of the IL-1β, IL-6, and IL-8 in cell culture supernatants were measured using ELISA kits (Thermo Fisher, MA, USA) according to the manufacturer’s procedure. The sensitivity of kits was 2 pg/ml.

### Measurements of transepithelial electrical resistance (TEER)

TEER measurements were performed using Millicell ERS-2 Voltohmmeter (Merck Millipore, Burlington, MA, USA) according to the manufacturer’s protocol. Two hundred microliters of PBS were added to the upper chamber in the cell culture system. The ohmic resistance of a blank (culture insert without cells) was evaluated for each experiment’s measurement and was subtracted from the total resistance of the sample.

### Flow cytometry analysis

For epithelial FACS measurements, cells from the upper chamber (ALI cultured epithelial cells alone or co-cultured with moMφs or/and moDCs) were evaluated. The cells were stained with antibodies against CD326, CD45, EGFR, mucin 1 (MUC1), TGF-β1, β-tubulin (BD Biosciences, San Jose, CA, USA), IL-33 receptor (ST2) (Biotechne, R&D Systems, MN, USA) and analyzed using the FACSCelesta instrument (BD Biosciences, San Jose, CA, USA). A detailed protocol of FACS analysis is described in the supplementary file, the characterization of fluorochrome-conjugated antibodies used for epithelial cell staining is shown in Table S2.

The epithelial cell subpopulations were defined as follows:

- Epithelial cells: CD45 − CD326 +
- Epithelial cells with secretory phenotype: CD45 − CD326 + MUC1 + β-Tubulin − 
- Epithelial cells with ciliary phenotype: CD45 − CD326 + MUC1 − β-Tubulin +. 

The proportions of positive cells for EGFR, TGF-β1 and ST2 were presented as a percentage in the epithelial gate or subpopulation of undefined epithelial cells, epithelial cells with secretory phenotype, and epithelial cells with ciliated phenotype gates.

### Statistical analysis

Statistical analysis was performed with the use of the Statistica 13.3 software package (StatSoft Inc., OK, USA). The Kruskal–Wallis test was used to assess differences between continuous variables in the three study groups. The Mann–Whitney U test was applied for pairwise comparisons. Pearson Chi-square test was used to compare intergroup differences in the categorical variables. Results are given as a median and interquartile range (IQR). Differences were considered statistically significant at *p* < 0.05.

### Statement of ethics

All procedures performed in this study were in accordance with the ethical standards of the institutional and/or national research committee and with the 1964 Helsinki Declaration and its later amendments or comparable ethical standards. This work has received approval for research ethics from Medical University of Warsaw Review Board (KB/37/2020) and a proof/certificate of approval is available upon request.

## Results

### The effect of UPM on cytokines’ mRNA expression in epithelial cells

**Figure 2:**
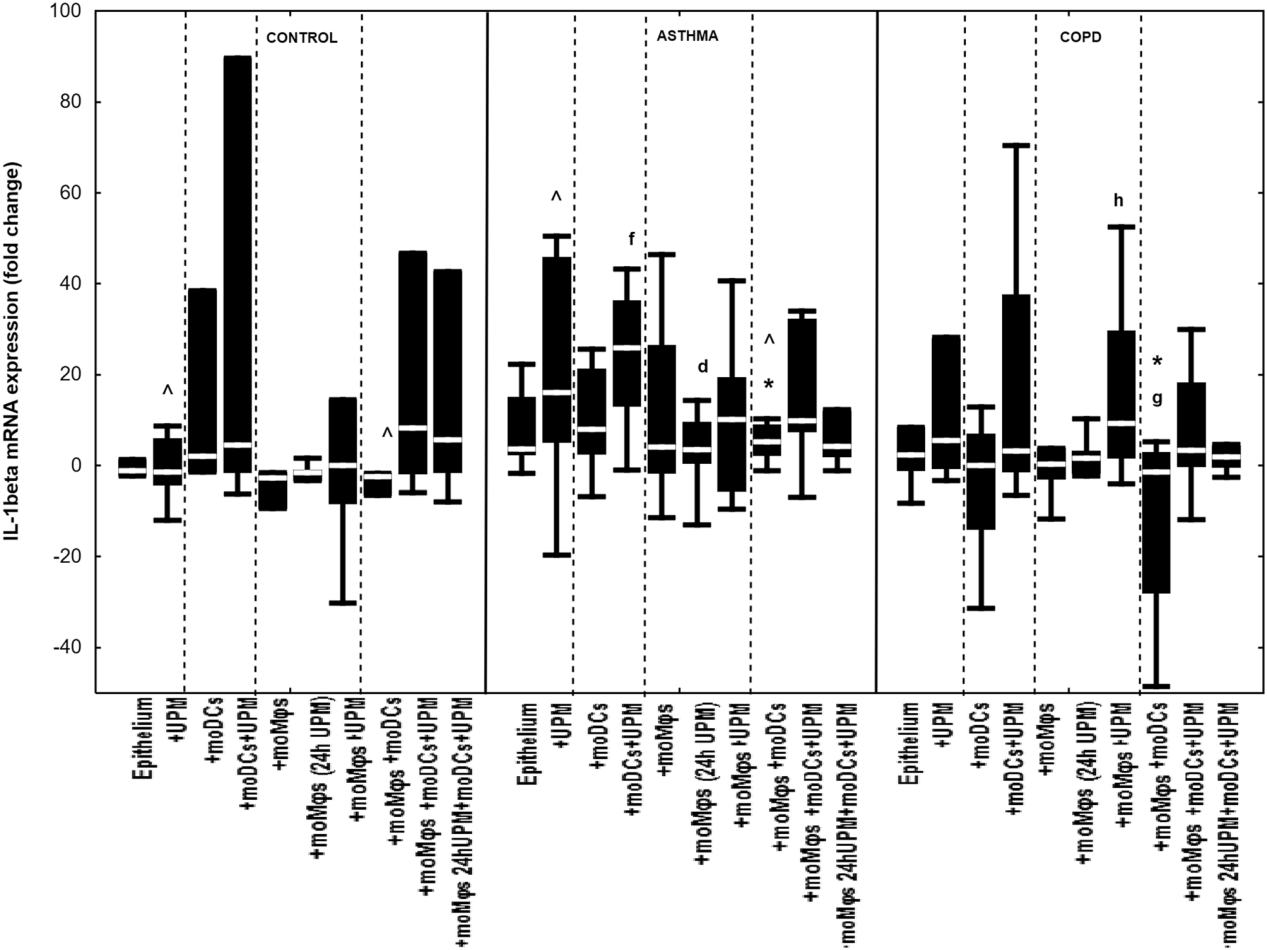
IL-1β mRNA expression in air–liquid interface (ALI) after 24 h UPM exposure in multi co-cultures in control subjects, asthma and COPD patients. The data are shown as non-outlier range (whiskers), interquartile range (box) and median (line); *p* value calculated using Mann–Whitney U test. The *p* value  < 0.05 in comparison to: a—epithelium, b—epithelium + UPM, c—epithelium + moDCs, d—epithelium + moDCs + UPM, e—epithelium + moMφs, f—epithelium + moMφs (24 h UPM), g—epithelium + moMφs + UPM, h—epithelium + moMφs + moDCs, i—epithelium + moMφs + moDCs + UPM, j—epithelium + moMφs (24hUMP) + moDCs + UPM; ^ asthma versus control, # COPD versus control, *asthma versus COPD.

**Figure 3:**
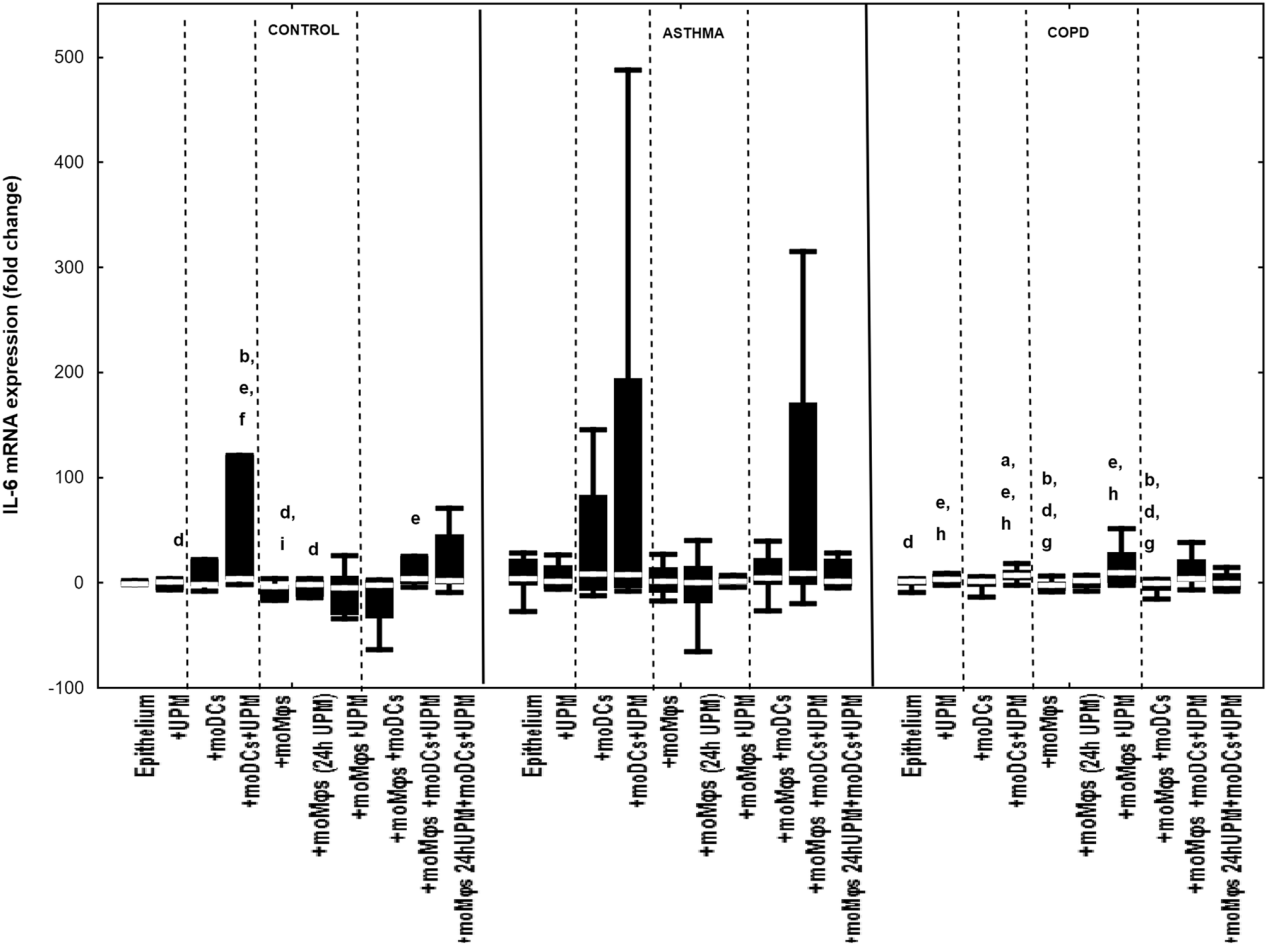
IL-6 mRNA expression in air–liquid interface (ALI) after 24 h UPM exposure in multi co-culture schemes in control subjects, asthma and COPD patients. The data are shown as non-outlier range (whiskers), interquartile range (box) and median (line); *p* value calculated using Mann–Whitney U test. The *p* value  < 0.05 in comparison to: a—epithelium, b—epithelium + UPM, c—epithelium + moDCs, d—epithelium + moDCs + UPM, e—epithelium + moMφs, f—epithelium + moMφs (24 h UPM), g—epithelium + moMφs + UPM, h—epithelium + moMφs + moDCs, i—epithelium + moMφs + moDCs + UPM, j—epithelium + moMφs (24hUMP) + moDCs + UPM; ^ asthma versus control, # COPD versus control, *asthma versus COPD.

**Figure 4:**
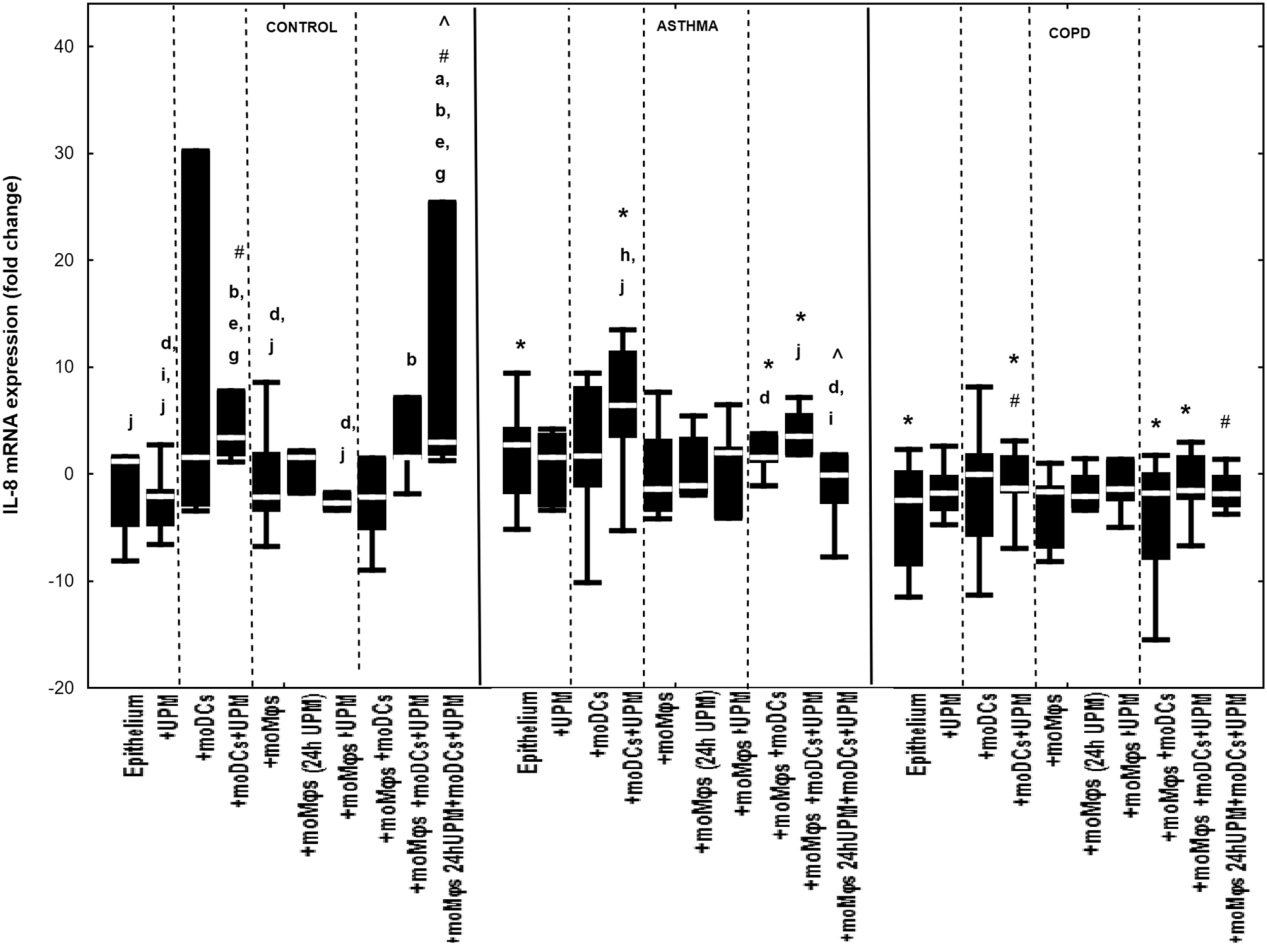
IL-8 mRNA expression in air–liquid interface (ALI) after 24 h UPM exposure in multi co-culture schemes in control subjects, asthma and COPD patients. The data are shown as non-outlier range (whiskers), interquartile range (box) and median (line); *p* value calculated using Mann–Whitney U test. The *p* value  < 0.05 in comparison to: a—epithelium, b—epithelium + UPM, c—epithelium + moDCs, d—epithelium + moDCs + UPM, e—epithelium + moMφs, f—epithelium + moMφs (24 h UPM), g—epithelium + moMφs + UPM, h—epithelium + moMφs + moDCs, i—epithelium + moMφs + moDCs + UPM, j—epithelium + moMφs (24hUMP) + moDCs + UPM; ^ asthma versus control, # COPD versus control, *asthma versus COPD.

**Figure 5:**
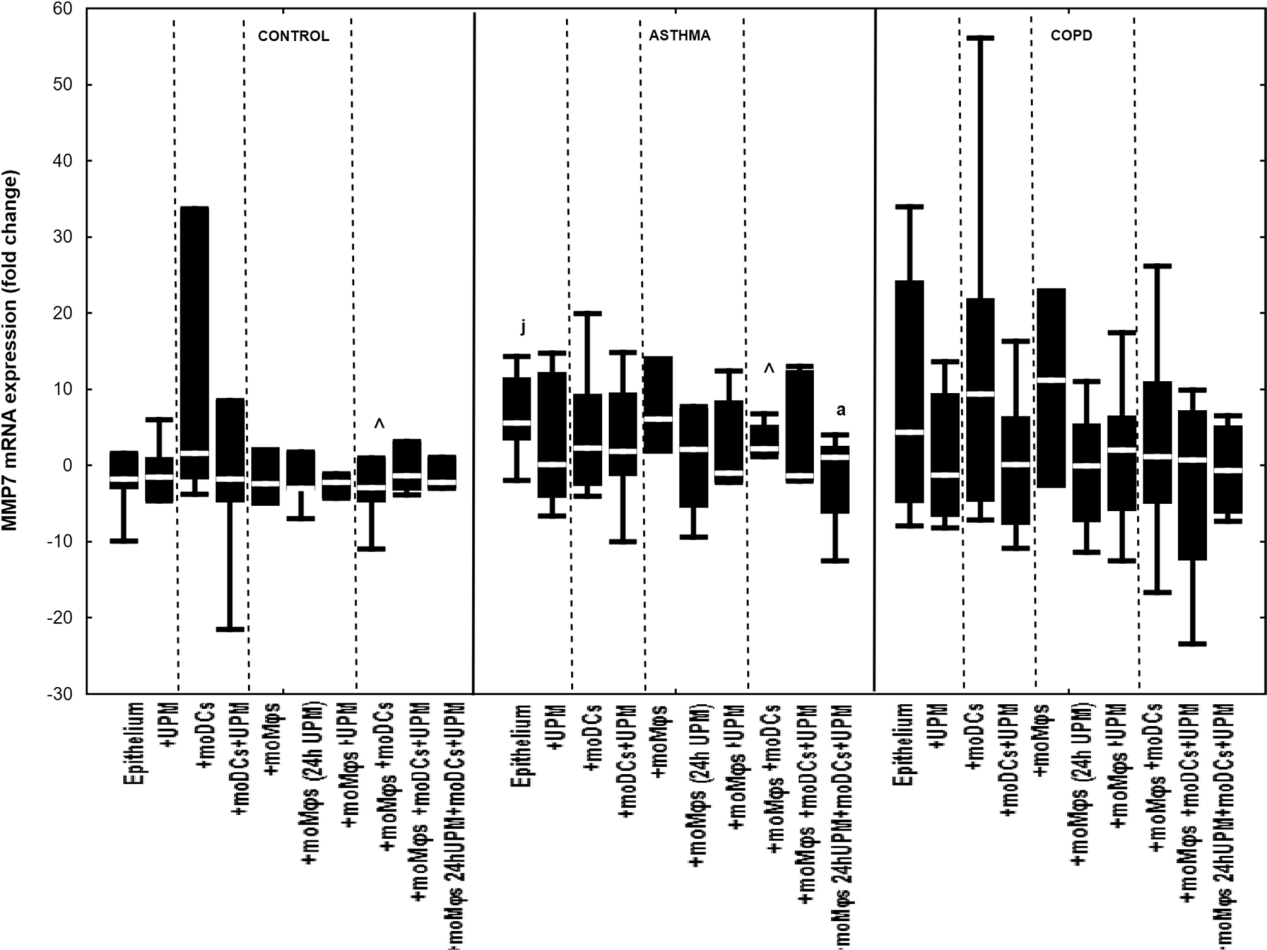
MMP7 mRNA expression in air–liquid interface (ALI) after 24 h UPM exposure in multi co-culture schemes in control subjects, asthma and COPD patients. The data are shown as non-outlier range (whiskers), interquartile range (box) and median (line); *p* value calculated using Mann–Whitney U test. The *p* value  < 0.05 in comparison to: a—epithelium, b—epithelium + UPM, c—epithelium + moDCs, d—epithelium + moDCs + UPM, e—epithelium + moMφs, f—epithelium + moMφs (24 h UPM), g—epithelium + moMφs + UPM, h—epithelium + moMφs + moDCs, i—epithelium + moMφs + moDCs + UPM, j—epithelium + moMφs (24hUMP) + moDCs + UPM; ^ asthma versus control, # COPD versus control, *asthma versus COPD.

**Figure 6:**
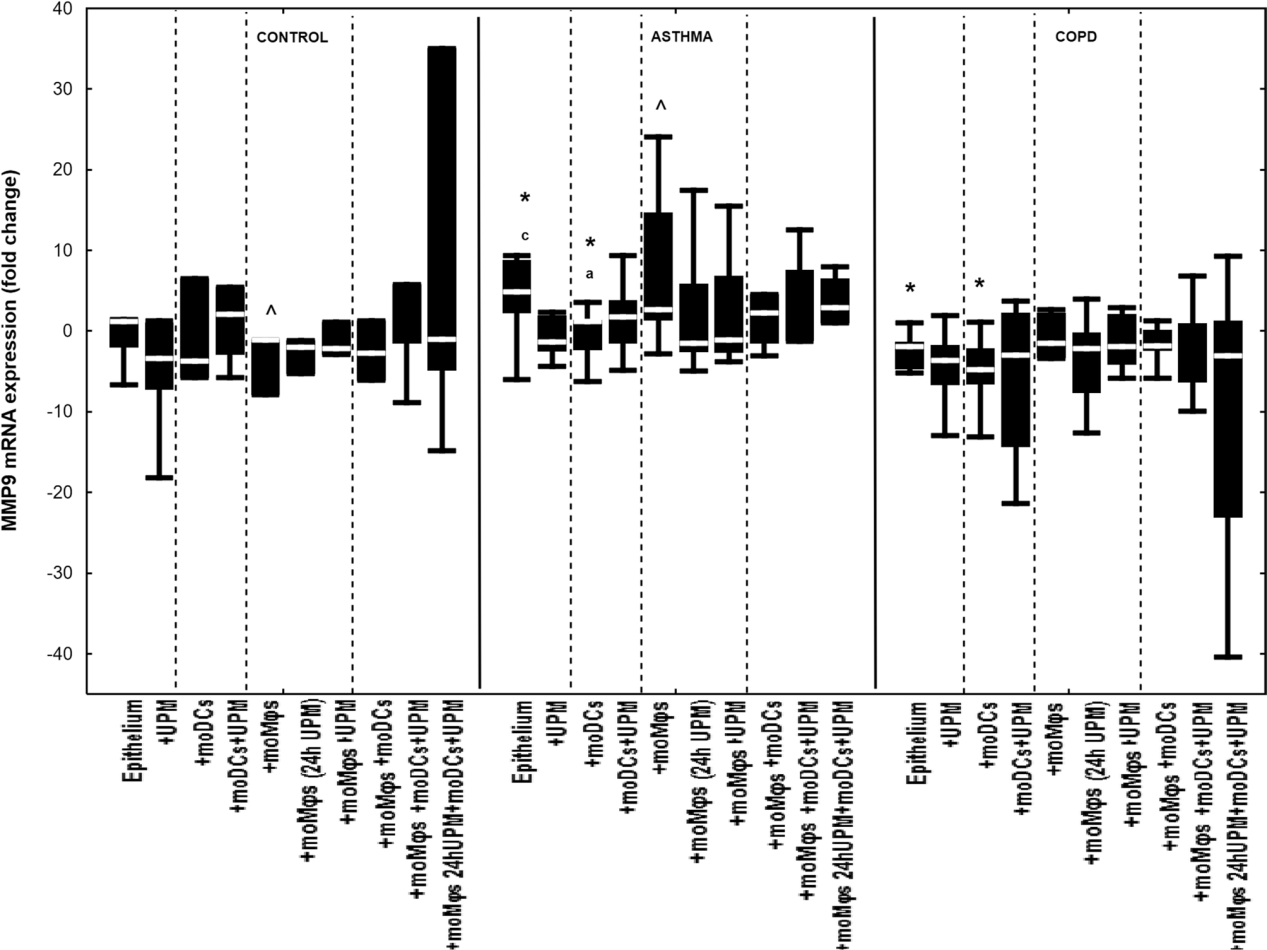
MMP9 mRNA expression in air–liquid interface (ALI) after 24 h UPM exposure in multi co-culture schemes in control subjects, asthma and COPD patients. The data are shown as non-outlier range (whiskers), interquartile range (box) and median (line); *p* value calculated using Mann–Whitney U test. The *p* value  < 0.05 in comparison to: a—epithelium, b—epithelium + UPM, c—epithelium + moDCs, d—epithelium + moDCs + UPM, e—epithelium + moMφs, f—epithelium + moMφs (24 h UPM), g—epithelium + moMφs + UPM, h—epithelium + moMφs + moDCs, i—epithelium + moMφs + moDCs + UPM, j—epithelium + moMφs (24hUMP) + moDCs + UPM; ^ asthma versus control, # COPD versus control, *asthma versus COPD.

**Figure 7:**
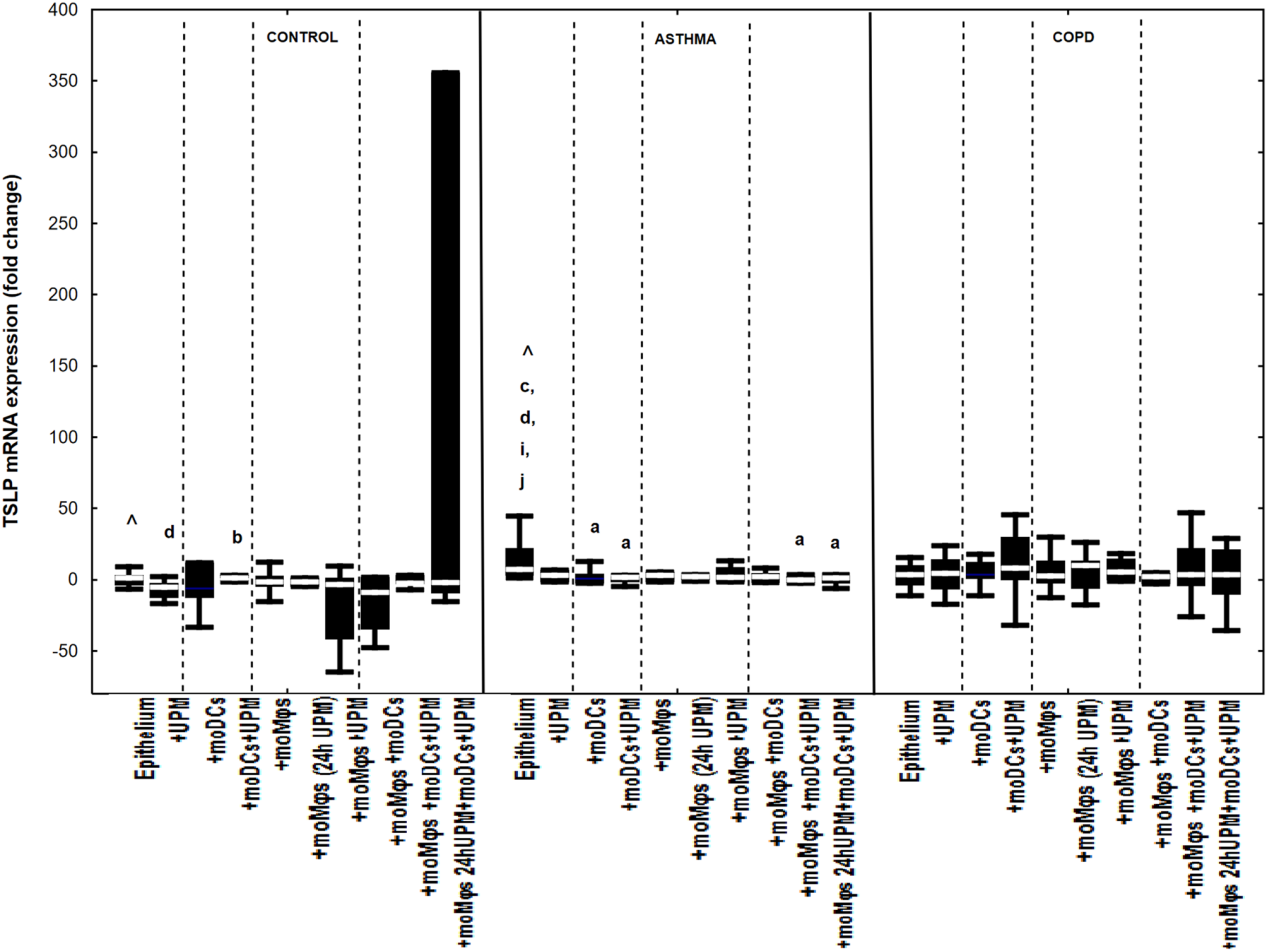
TSLP mRNA expression in air–liquid interface (ALI) after 24 h UPM exposure in multi co-culture schemes in control subjects, asthma and COPD patients. The data are shown as non-outlier range (whiskers), interquartile range (box) and median (line); *p* value calculated using Mann–Whitney U test. The *p* value  < 0.05 in comparison to: a—epithelium, b—epithelium + UPM, c—epithelium + moDCs, d—epithelium + moDCs + UPM, e—epithelium + moMφs, f—epithelium + moMφs (24 h UPM), g—epithelium + moMφs + UPM, h—epithelium + moMφs + moDCs, i—epithelium + moMφs + moDCs + UPM, j—epithelium + moMφs (24hUMP) + moDCs + UPM; ^ asthma versus control, # COPD versus control, *asthma versus COPD.

**Figure 8:**
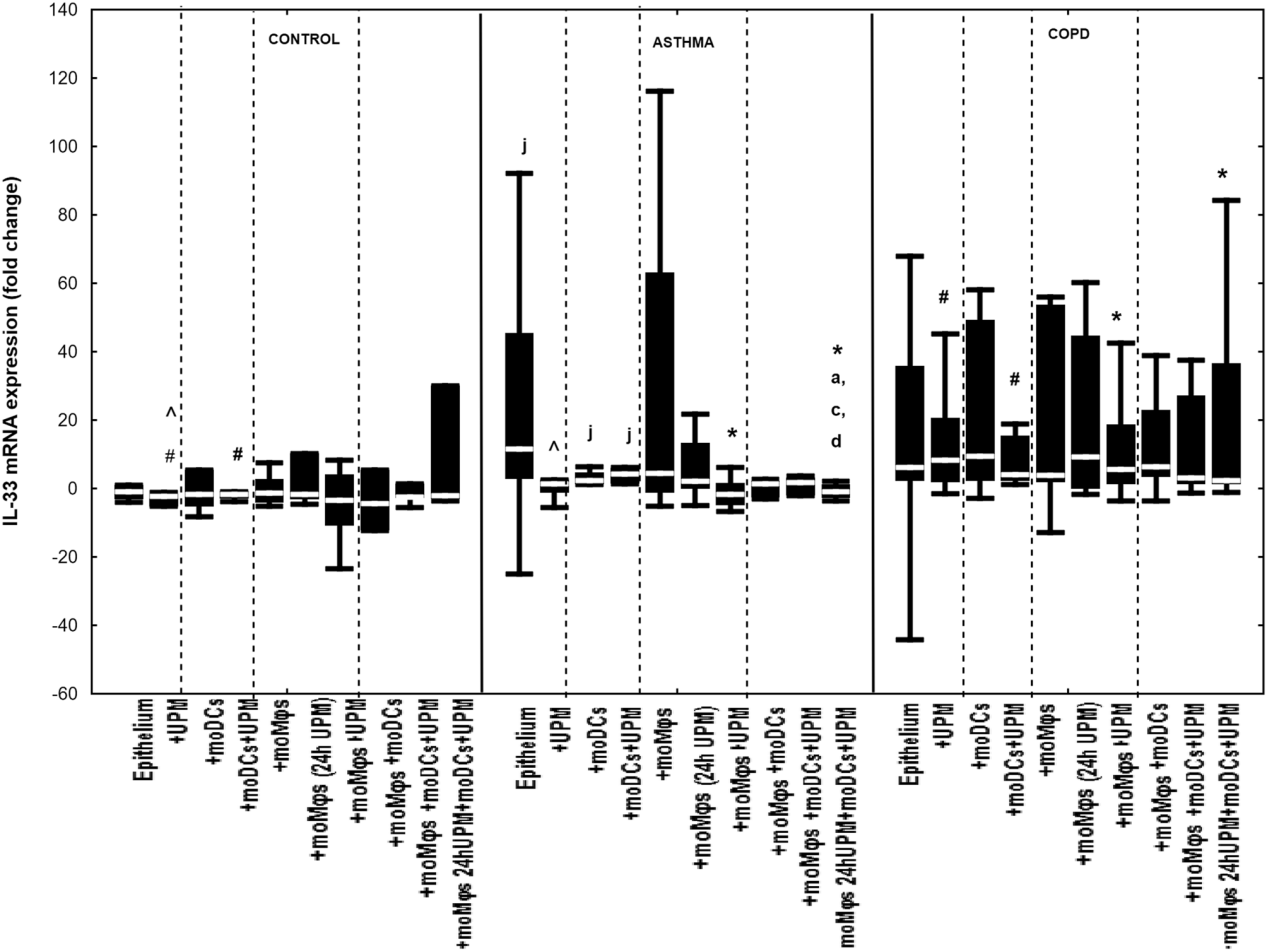
IL-33 mRNA expression in air–liquid interface (ALI) after 24 h UPM exposure in multi co-culture schemes in control subjects, asthma and COPD patients. The data are shown as non-outlier range (whiskers), interquartile range (box) and median (line); *p* value calculated using Mann–Whitney U test. The *p* value  < 0.05 in comparison to: a—epithelium, b—epithelium + UPM, c—epithelium + moDCs, d—epithelium + moDCs + UPM, e—epithelium + moMφs, f—epithelium + moMφs (24 h UPM), g—epithelium + moMφs + UPM, h—epithelium + moMφs + moDCs, i—epithelium + moMφs + moDCs + UPM, j—epithelium + moMφs (24hUMP) + moDCs + UPM; ^ asthma versus control, # COPD versus control, *asthma versus COPD.

Co-cultivation of epithelial cells with moDCs and/or moMφs without UPM treatment did not change mRNA expression for IL-1β, IL-6, IL-8, MMP7, MMP9, TSLP, and IL-33 in evaluated groups, except for decreased MMP9 and TSLP mRNA expression in asthmatic epithelium co-cultivated with moDCs compared to epithelial cells alone (Figs. 2, 3, 4, 5, 6, 7, 8). The exact medians and IQR values of the fold change of cytokine mRNA expression are shown in Table S3.

24 h cultivation of moDCs in epithelial growth medium did not affect cell viability (84.6% (79.2–89.7%) versus 93.9% (92.6–95.6%), *p* = 0.114 of moDCs cultured in DC and epithelial dedicated medium, respectively) as well as the distribution of viable, apoptotic and necrotic cells within these groups.

Exposure to UPM increased IL-1β mRNA expression in epithelium/moDCs di-cultures (25.9 fold change (13.0–36.2 fold change)) compared to epithelial/moMφs (UPM 24 h) co-cultures (3.5 fold change (0.5–9.5 fold change), *p* = 0.015) in asthma, and in epithelial/moMφs (9.3 fold change (1.6–29.7 fold change) compared to unstimulated triple co-cultures (− 1.5 fold change (− 28.1 to 2.9 fold change), *p* = 0.02) in COPD (Fig. 2).

The comparison between groups showed that the highest changes in IL-1β mRNA expression after UPM exposure were observed in asthmatic epithelial cells alone (16.1 fold change (5.1–45.8 fold change) compared to analogous cell cultures in controls (− 1.4 fold change (− 4.3 to 6.0 fold change), *p* = 0.04) (Fig. 2).

The greatest changes in IL-6 mRNA expression after UPM stimulation were observed in controls in epithelium/moDCs di-cultures (4.0 fold change (3.5–121.1 fold change) compared to UPM treated epithelium alone (1.1 fold change (− 6.5 to 2.6 fold change), *p* = 0.03), unstimulated epithelium/moMφs co-cultures (− 3.7 fold change (− 16.7 to − 2.0 fold change), *p* = 0.004) and epithelium/moMφs (UPM24h) co-cultures (− 0.2 fold change (− 13.0 to 2.3 fold change), *p* = 0.01). UPM exposure upgraded IL-6 mRNA expression in triple co-cultures (3.6 fold change (− 1.2 to 25.1 fold change)) compared to unstimulated epithelium/moMφs, (*p* = 0.035) in controls. A similar pattern of changes after UPM treatment was noted in COPD with additional increased IL-6 mRNA expression in epithelium/moMφs di-cultures (9.8 fold change (0.4 to 20.1 fold change)) compared to unstimulated epithelium/moMφs di-cultures (− 2.4 fold change (− 7.0 to 1.1 fold change)) (*p* = 0.02) (Fig. 3).

The changes in expression of IL-6 mRNA after UPM stimulation did not differ between control, asthma and COPD groups (Fig. 3).

In control group, UPM exposure increased IL-8 mRNA expression in epithelium/moDCs di-cultures (3.4 fold change (1.7–7.8 fold change)) compared to UPM treated epithelial cells alone (− 2.1 fold change (− 4.8 to − 1.4 fold change), *p* = 0.002), unstimulated (− 2.2 fold change (− 3.5 to 2.0 fold change), *p* = 0.04) and UPM treated epithelial/moMφs co-cultures (− 2.7 fold change (− 3.4 to 1.7 fold change), *p* = 0.04). UPM upgraded IL-8 mRNA expression in triple co-cultures, with the highest changes in triple co-cultures with moMφs (24 h UPM) (3.0 fold change (1.7–25.4 fold change)) in controls. In asthma similarly to control group the biggest changes in IL-8 mRNA expression after UPM exposure was found in epithelial/moDCs co-cultures (6.4 fold change (3.4–11.5 fold change) and in triple co-cultures (3.5 fold change (1.8–5.7 fold change). No significant changes were observed in IL-8 mRNA expression in UPM treated cells in COPD (Fig. 4).

The asthmatic epithelium/moDCs co-cultures (6.4 fold change (3.4 to 11.5 fold change)) as well as triple-co-cultures (3.5 fold change (1.8 to 5.7 fold change)) were characterised by the most substantial changes in IL-8 mRNA expression after UPM exposure compared to COPD (− 1.3 fold change (− 1.8 to 1.7 fold change), *p* = 0.01; (− 1.5 fold change (− 2.4 to 1.7 fold change), *p* = 0.04, for epithelial/moDCs and triple co-cultures, respectively) (Fig. 4).

UPM stimulation decreased MMP7 mRNA expression in triple-co-cultures with moMφs (UPM 24 h) (1.0 fold change (− 6.3 to 2.6 fold change)) compared to untreated epithelial cells alone (5.6 fold change (3.3 to 11.5 fold change), *p* = 0.049) in the asthma group. UPM treatment did not or slightly impact MMP7 mRNA expression in control and COPD group. (Fig. 5).

UPM exposure did not change MMP9 mRNA expression in control, asthma or COPD group in any of the epithelial co-cultures (Fig. 6).

The most pronounced effect concerning changes in TSLP mRNA expression after UPM exposure was noted in asthma group. UPM treatment decreased TSLP mRNA expression in epithelial/moDCs co-cultures (1.8 fold change (− 1.3 to 2.4 fold change)), triple co-cultures (− 0.1 fold change (− 2.0 to 2.8 fold change) and triple co-cultures with moMφs (24 UPM) (1.3 fold change (− 2.5 to 2.8 fold change)) compared to unexposed epithelial cells alone (7.1 fold change (2.9 to 45.5 fold change), *p* = 0.02, *p* = 0.02, *p* = 0.01, respectively). No changes for TSLP mRNA expression after UPM exposure were observed in the COPD group (Fig. 7).

MMP7, MMP9 and TSLP presented the same pattern of mRNA expression changes after UPM exposure in all evaluated groups (Fig. 5, 6, 7).

We found no significant alterations of IL-33 mRNA expression in the epithelium after UPM stimulation in the control or COPD group. In asthma, UPM exposure decreased IL-33 mRNA expression in triple co-cultures with moMφs (24 h UPM) (–1.1 fold change (− 2.8 to 1.2 fold change)) compared to unstimulated epithelial cells alone (11.6 fold change (2.9 to 45.5 fold change), *p* = 0.01), unstimulated epithelium/moDCs di-cultures ( 2.3 fold change (1.3 to 4.7 fold change), *p* = 0.04) and UPM exposed epithelium/moDCs di-cultures (4.2 fold change (1.5 to 6.0 fold change), *p* = 0.02) (Fig. 8).

The asthmatics epithelial cells alone after UPM treatment expressed the highest IL-33 mRNA level compared to controls (*p* = 0.02). However, the most profound changes after UPM stimulation for IL-33 mRNA expression was found for COPD epithelial/moDCs co-cultures (compared to control, *p* = 0.02) epithelial/ moMφs co-cultures (*p* = 0.03) and triple co-cultures (*p* = 0.01) (compared to asthma) (Fig. 8).

### The effect of UPM on cytokine proteins’ secretion by epithelial cells

**Figure 9:**
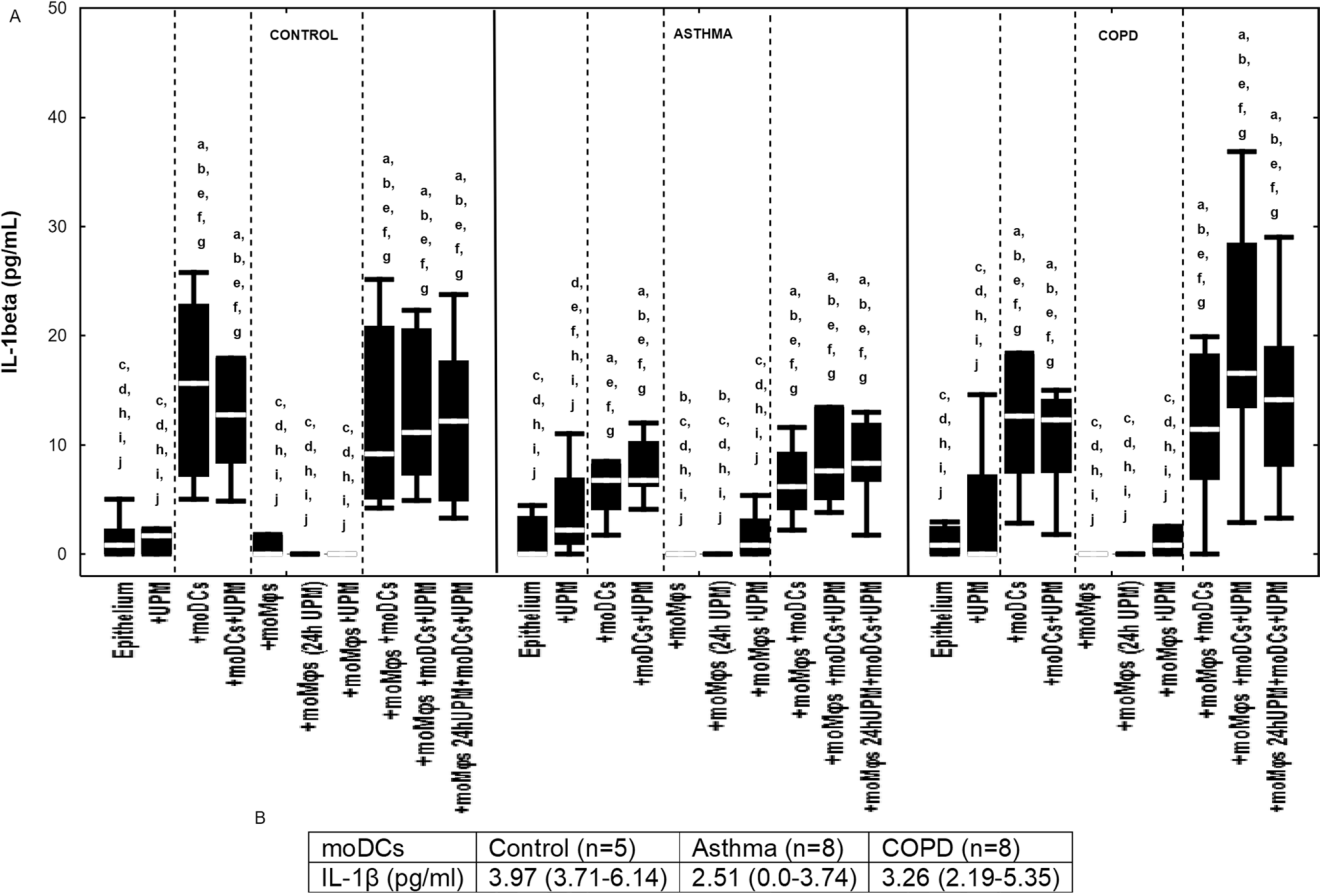
(**A**) IL-1β secretion by air–liquid interface (ALI) cultured nasal epithelium after 24 h UPM exposure in multi co-culture schemes in control subjects, asthma and COPD patients. (**B**) IL-1β levels produced by moDCs cultured alone are shown in table. The data are shown as non-outlier range (whiskers), interquartile range (box) and median (line). The exact *p* value calculated after Mann–Whitney U test are shown in supplementary file (Table S5, Table S6 and Table S7). The *p* value  < 0.05 in comparison to: a—epithelium, b—epithelium + UPM, c—epithelium + moDCs, d—epithelium + moDCs + UPM, e—epithelium + moMφs, f—epithelium + moMφs (24 h UPM), g- epithelium + moMφs + UPM, h—epithelium + moMφs + moDCs, i—epithelium + moMφs + moDCs + UPM, j—epithelium + moMφs (24hUMP) + moDCs + UPM; ^ asthma versus control, # COPD versus control, *asthma versus COPD.

**Figure 10:**
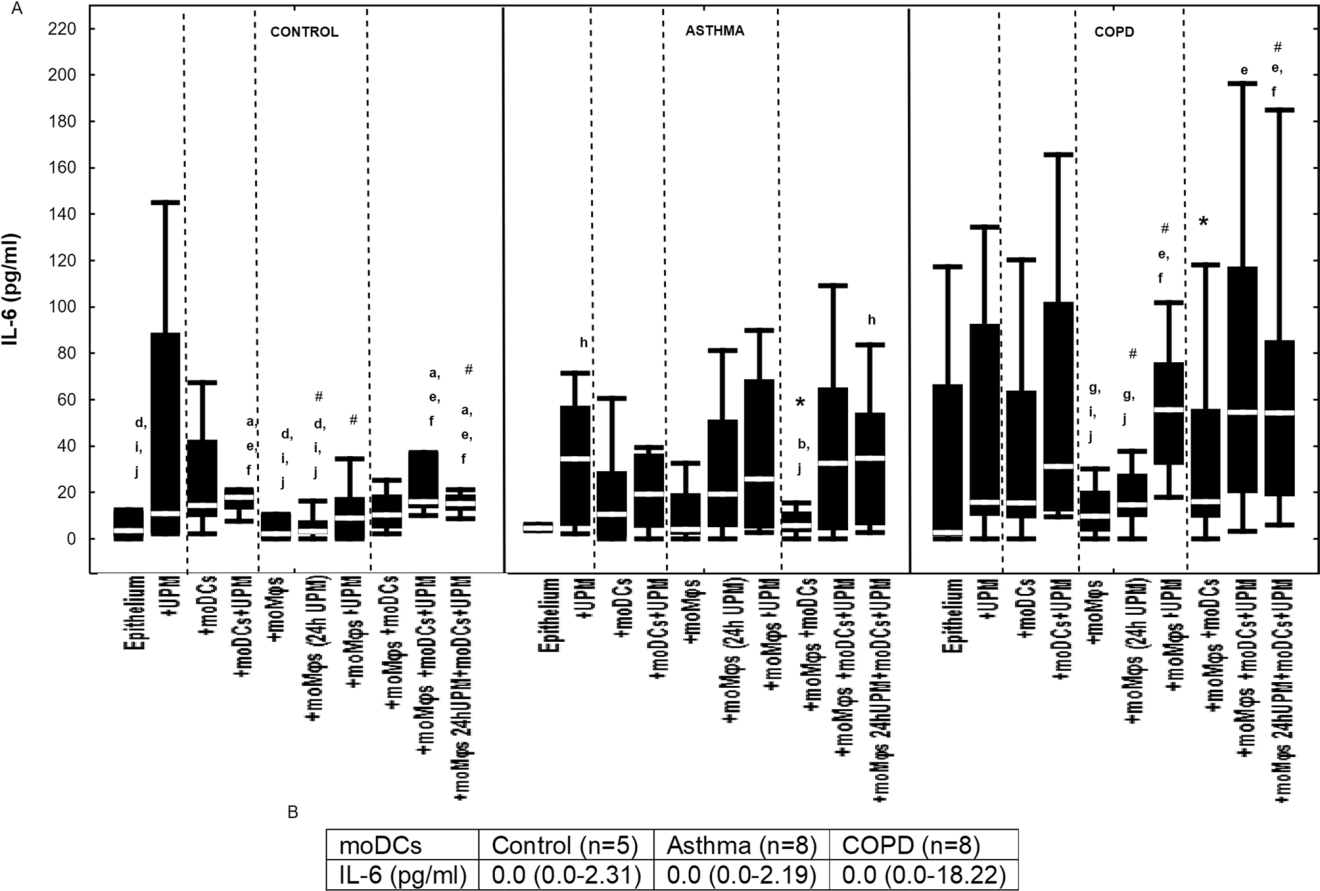
(**A**) IL-6 secretion by air–liquid interface (ALI) cultured nasal epithelium after 24 h UPM exposure in multi co-culture schemes in control subjects, asthma and COPD patients. (**B**) IL-6 levels produced by moDCs cultured alone are shown in table. The data are shown as non-outlier range (whiskers), interquartile range (box) and median (line); *p* value calculated using Mann–Whitney U test. The *p* value  < 0.05 in comparison to: a—epithelium, b—epithelium + UPM, c—epithelium + moDCs, d—epithelium + moDCs + UPM, e—epithelium + moMφs, f—epithelium + moMφs (24 h UPM), g- epithelium + moMφs + UPM, h—epithelium + moMφs + moDCs, i—epithelium + moMφs + moDCs + UPM, j—epithelium + moMφs (24hUMP) + moDCs + UPM; ^ asthma versus control, # COPD versus control, *asthma versus COPD.

**Figure 11:**
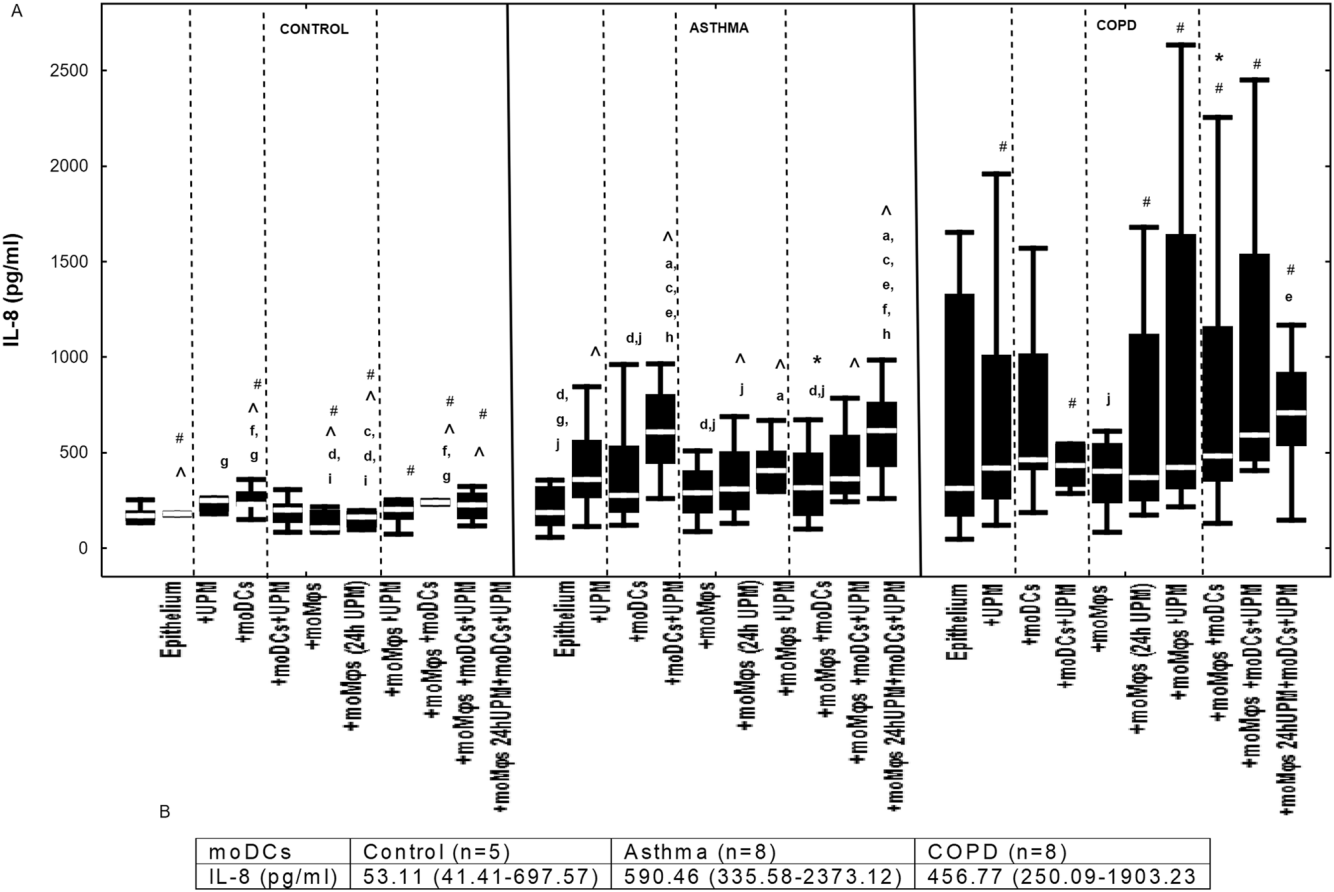
(**A**) IL-8 secretion by air–liquid interface (ALI) cultured nasal epithelium after 24 h UPM exposure in multi co-culture schemes in control subjects, asthma and COPD patients. (**B**) IL-8 levels produced by moDCs cultured alone are shown in table. The data are shown as non-outlier range (whiskers), interquartile range (box) and median (line); *p* value calculated using Mann–Whitney U test. The *p* value  < 0.05 in comparison to: a—epithelium, b—epithelium + UPM, c—epithelium + moDCs, d—epithelium + moDCs + UPM, e—epithelium + moMφs, f—epithelium + moMφs (24 h UPM), g- epithelium + moMφs + UPM, h—epithelium + moMφs + moDCs, i—epithelium + moMφs + moDCs + UPM, j—epithelium + moMφs (24hUMP) + moDCs + UPM; ^ asthma versus control, # COPD versus control, *asthma versus COPD.

Nasal epithelium cultured together with moDCs (both di- and tri-co-cultures) without UPM stimulation produced a significantly higher amount of IL-1β in all studied groups (Fig. 9). Co-cultivation of epithelial cells with moDCs and/or moMφs without UPM did not change IL-6 or IL-8 protein secretion in the control, asthma, and COPD groups (Figs. 10, 11). The exact medians and IQR values of the fold change of cytokine protein levels are shown in Table S4.

UPM stimulation did not alter the IL-1β level in epithelial cultures compared to corresponding unstimulated control cells, asthma and COPD patients (Fig. 9). Detailed comparisons of the IL-1β protein level in control, asthma and COPD are presented in Tables S5, S6, and S7. UPM exposure impacted IL-1β production in similar pattern in all groups.

We observed a few variations in IL-6 secretion after UPM exposure in the controls, including slightly higher IL-6 levels in epithelial/moDCs (18.0 pg/ml (12.74–21.14 pg/ml)) and triple-co-cultures (with UPM 24 h moMφs) (15.20 pg/ml (12.29–19.09 pg/ml)) compared to epithelial cells alone (3.44 pg/ml (1.13–11.94 pg/ml)) (*p* = 0.022 and *p* = 0.01, respectively) or epithelium co-cultivated with moMφs (2.16 pg/ml (0.0–10.46 pg/ml)) (*p* = 0.026 and *p* = 0.022, respectively). In asthma, the highest IL-6 production after UPM treatment was noted in epithelial cells alone (34.33 pg/ml (5.77–57.26 pg/ml)) and in triple-co-cultures with moMφs (24 h UMP) (34.60 pg/ml (6.03–54.27 pg/ml)) compared to unstimulated triple co-cultures (5.63 pg/ml (2.94–11.74 pg/ml), *p* = 0.038 and *p* = 0.008, respectively). In COPD, an elevated level of IL-6 after UPM exposure was found in epithelial/moMφs di-cultures (55.58 pg/ml (31.92–76.04 pg/ml) compared to unstimulated epithelial/moMφs di-cultures (9.77 pg/ml (3.31–20.57 pg/ml)) (*p* = 0.021) and in triple co-cultures (54.50 pg/ml (19.81–117.37 pg/ml)) compared to unexposed epithelial/moMφs di-cultures (*p* = 0.049) (Fig. 10).

The highest amount of IL-6 after UPM stimulation was produced by epithelial/moMφs co-cultures (*p* = 0.005) and triple co-cultures (*p* = 0.004) in COPD group compared to controls (Fig. 10).

Upgraded IL-8 production after UPM stimulation was observed in triple co-cultures (238.23 pg/ml (229.59–247.95 pg/ml)) compared to epithelium/moMφs (24 h UPM) di-cultures (105.16 pg/ml (84.86–199.0 pg/ml)) (*p* = 0.017) in the control group. UPM exposure enhanced IL-8 secretion in epithelial/moDCs di-cultures (607.66 pg/ml (442.91–804.51 pg/ml)) in asthma and in triple-co-culture with moMφs (24 h UPM) (614.41 pg/ml (425.41–764.81 pg/ml)) compared to unexposed triple co-cultures (315.93 pg/ml (168.30–500.06 pg/ml)) (*p* = 0.013 and *p* = 0.003, respectively) and epithelial/moMφs co-cultures (288.43 pg/ml (182.33–405.59 pg/ml)) (*p* = 0.007 and *p* = 0.002, respectively) in asthma. In COPD, UPM exposure increased IL-8 production in triple-co-culture with moMφs (24 h UPM) (708.14 pg/ml (533.57–921.53 pg/ml)) compared to epithelium/moMφs co-cultures without UPM treatment (403.1 pg/ml (237.33–549.72 pg/ml)) (*p* = 0.040). However, due to the high production of IL-8 by moDCs in asthma and COPD alone (*p* value > 0.05), the evaluation of IL-8 secretion by di-and triple co-cultures with moDCs should be interpreted carefully (Fig. 11).

Asthmatic and COPD epithelium produced more IL-8 protein after UPM treatment than epithelium from control group, especially in asthma epithelial/moDCs co-cultures (*p* = 0.003) and COPD triple co-cultures (*p* = 0.0007) compared to controls (Fig. 11).

### The effect of UPM on cytokine mRNA expression and proteins’ secretion by moMφs

UPM exposure did not change moMφs viability (76.8% (76.1–83.7%) versus 80.2% (75.1–84.7%), *p* = 0.886 for untreated and UPM treated moMφs, respectively), but it shifted necrotic and apoptotic cell distribution within the group from 65.2% (54.3–80.4%) to 34% (31.6–37.1%), *p* = 0.03 for necrotic cells, and from 5.04% (4.1–5.1%) to 36.3% (32.2–38.8%) for apoptotic cells in untreated and UPM exposed moMφs, respectively.

The mRNA expression of inflammatory mediators, as well as the levels of IL-1β, IL-6 and IL-8 after UPM exposure in control, asthma, and COPD moMφs are presented in Tables 3 and 4. We observed decreased mRNA expression of TSLP in asthma and decreased expression of IL-6 mRNA COPD in moMφs after the exposure to UPM (Table 3). The moMφs in control group secreted increased level of IL-1β protein after the UPM treatment (Table 4).

**Table 3:** mRNA expression of IL-1β, IL-6, IL-8, MMP7, MMP9, IL-33 and TSLP in moMφs of control, asthma and COPD patients with and without UPM treatment.

mRNA expression (fold change)	moMφs	moMφs + UPM	*p* value
**Control (n = 7)**			
IL-1β	− 1.2 (− 2.4 to 3.1)	− 1.6 (− 1.9 to 2.0)	0.71
IL-6	− 1.3 (− 1.4 to 1.1)	0.0 (− 1.5 to 1.6)	0.93
IL-8	1.3 (− 2.9 to 2.0)	− 1.0 (− 1.9 to 2.1)	1.00
MMP7	− 2.3 (− 3.0 to 6.3)	− 1.4 (− 3.4 to 5.4)	1.00
MMP9	1.3 (− 2.4 to 1.6)	− 1.2 (− 1.4 to 1.2)	0.53
TSLP	− 1.1 (− 3.9 to 1.7)	− 1.3 (− 6.6 to − 1.0)	0.84
IL-33	1.4 (− 2.5 to 3.8)	1.7 (− 8.3 to 4.0)	0.95
**Asthma (n = 8)**			
IL-1β	0.0 (− 1.7 to 1.8)	1.1 (− 2.2 to 2.3)	1.00
IL-6	7.5 (− 7.6 to 46.1)	1.1 (− 2.1 to 5.4)	0.90
IL-8	− 1.3 (− 1.8 to 2.1)	1.1 (− 1.8 to 1.9)	0.85
MMP7	1.2 (− 1.1 to 1.9)	− 1.4 (− 2.3 to 3.2)	0.96
MMP9	0.1 (− 1.5 to 1.4)	1.8 (− 3.6 to 2.6)	0.28
TSLP	1.7 (− 1.4 to 1.8)	− 34.2 (− 54.6 to − 5.7)	0.049
IL-33	− 0.2 (− 2.9 to 1.3)	− 1.6 (− 1.9 to 3.0)	0.95
**COPD (n = 7)**			
IL-1β	1.3 (− 1.5 to 1.6)	1.1 (− 2.0 to 1.6)	0.71
IL-6	1.5 (− 1.6 to 2.4)	0.0 (0.0 to 0.0)	0.001
IL-8	− 1.1 (− 1.6 to 2.0)	− 1.4 (− 4.2 to 1.1)	0.53
MMP7	− 1.6 (− 4.4 to 6.5)	− 1.4 (− 1.8 to 1.6)	0.90
MMP9	3.7 (− 1.4 to 7.0)	− 2.1 (− 2.6 to 3.0)	0.37
TSLP	0.3 (− 16.7 to 13.2)	− 2.3 (− 3.8 to 8.5)	0.89
IL-33	− 1.3 (− 2.0 to 4.6)	− 1.7 (− 11.7 to 3.0)	0.63

The results are presented as median and IQR.

**Table 4:** The level of IL-1β, IL-6, IL-8, secreted by moMφs of control, asthma and COPD patients with and without UPM treatment.

pg/ml	moMφs	moMφs + UPM	*p* value
**Control (n = 7)**			
IL-1β	0.0 (0.0–0.0)	1.5 (1.5–1.7)	0.04
IL-6	3.5 (0.0–10.1)	2.8 (0.0–14.0)	0.80
IL-8	812.4 (209.8–971.8)	979.6 (633.0–1101.3)	0.26
**Asthma (n = 8)**			
IL-1β	0.0 (0.0–0.0)	0.0 (0.0–0.0)	0.72
IL-6	0.0 (0.0–3.2)	0.0 (0.0–0.0)	0.80
IL-8	228.1 (92.8–716.5)	646.8 (39.7–1518.1)	0.72
**COPD (n = 7)**			
IL-1β	1.6 (0.0–2.9)	0.0 (0.0–2.6)	0.62
IL-6	0.0 (0.0–0.0)	0.0 (0.0–0.0)	0.71
IL-8	2340.0 (1422.7–2698.1)	1408.9 (950.9–1901.4)	0.05

The results are presented as median and IQR.

The comparison between groups revealed that moMφs after UPM stimulation produced higher amount of IL-1β protein in asthma group compared to controls (*p* = 0.004). The untreated moMφs from controls produced more IL-6 protein than moMφs from COPD patients (*p* = 0.03). On the other hand, the unstimulated moMφs in CODP group secreted the highest IL-8 protein level compared to both controls (*p* = 0.01) and asthma (*p* = 0.002) moMφs (Table 4).

### The effect of UPM on transepithelial electrical resistance (TEER) changes

**Figure 12:**
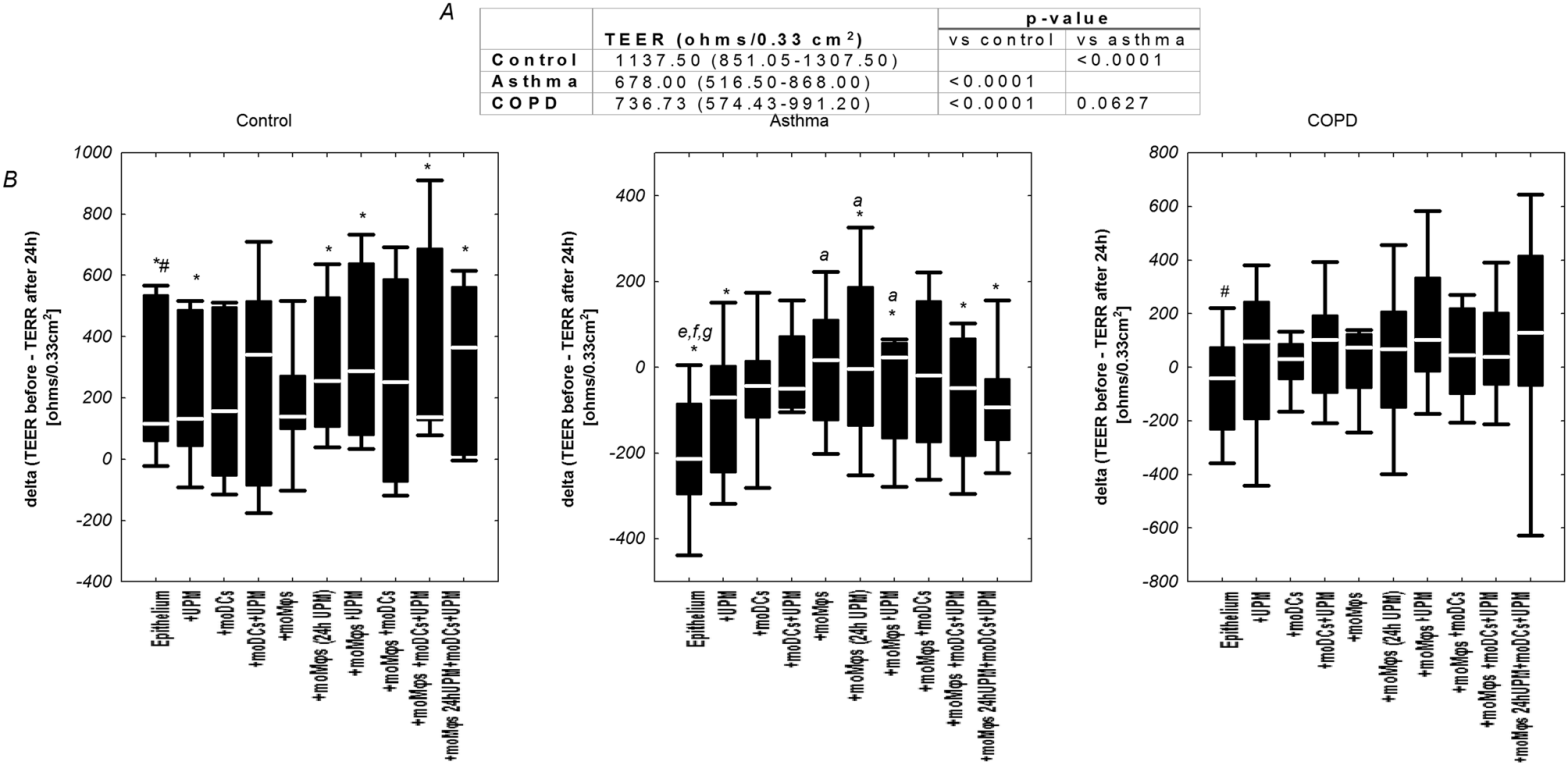
(**A**) The absolute TEER values of control, asthma, and COPD epithelial cells are shown in table. (**B**) The changes in trans epithelial electrical resistance (TEER) after 24 h of nasal epithelial cells from healthy donors, asthma and COPD cultured in air–liquid interface (ALI) conditions in multi co-culture models and UPM exposure. * p* value calculated using Mann–Whitney U test *p* value  < 0.05 in comparisons * control versus asthma, # control versus COPD; a—epithelium, b—epithelium + UPM, c—epithelium + moDCs, d—epithelium + moDCs + UPM, e—epithelium + moMφs, f—epithelium + moMφs (24 h UPM), g- epithelium + moMφs + UPM, h—epithelium + moMφs + moDCs, i—epithelium + moMφs + moDCs + UPM, j—epithelium + moMφs (24hUMP) + moDCs + UPM.

Asthma and COPD epithelial cells were characterized by lower TEER values compared to controls (Fig. 12A). A different pattern of TEER changes between mono, di- and triple co-cultures in controls and obstructive lung disease groups was observed: co-cultivation with moDCs and/or moMφs decreased TEER in controls but did not change TEER values in asthma and COPD (Figure S1, S2, S3). Asthma and control epithelial cells differed in terms of changes in TEER values after 24 h in most cultivation combinations, whereas such dependency was not observed in COPD, with the exception of unstimulated epithelial cells alone. Importantly, different TEER changes in epithelial co-cultures after 24 h were demonstrated: decreased in controls (∆TEER > 0), increased or without changes in asthma (∆TEER < 0) and without changes in COPD. Only in asthma ∆TEER of epithelial/moMφs (16.88 ∆TEER Ω/0.33 cm^2^ (− 123.13 to 109.88 ∆TEER Ω/0.33 cm^2^), epithelial/moMφs (24 h UPM) (− 3.13 ∆TEER Ω/0.33 cm^2^ (− 135.5 to 187.25 ∆TEER Ω/0.33 cm^2^), and epithelial/moMφs treated with UPM (23.0 ∆TEER Ω/0.33 cm^2^ (− 164.63 to 56.75 ∆TEER Ω/0.33 cm^2^) was increased compared to unexposed epithelial cells alone (− 213.78 ∆TEER Ω/0.33 cm^2^ (− 294.75 to (− 85.88) ∆TEER Ω/0.33 cm^2^) (*p* = 0.02, *p* = 0.04, *p* = 0.049, respectively) (Fig. 12B).

### The effect of UPM on the expression of EGFR, TGFβ and ST2 in epithelial cells

**Figure 13:**
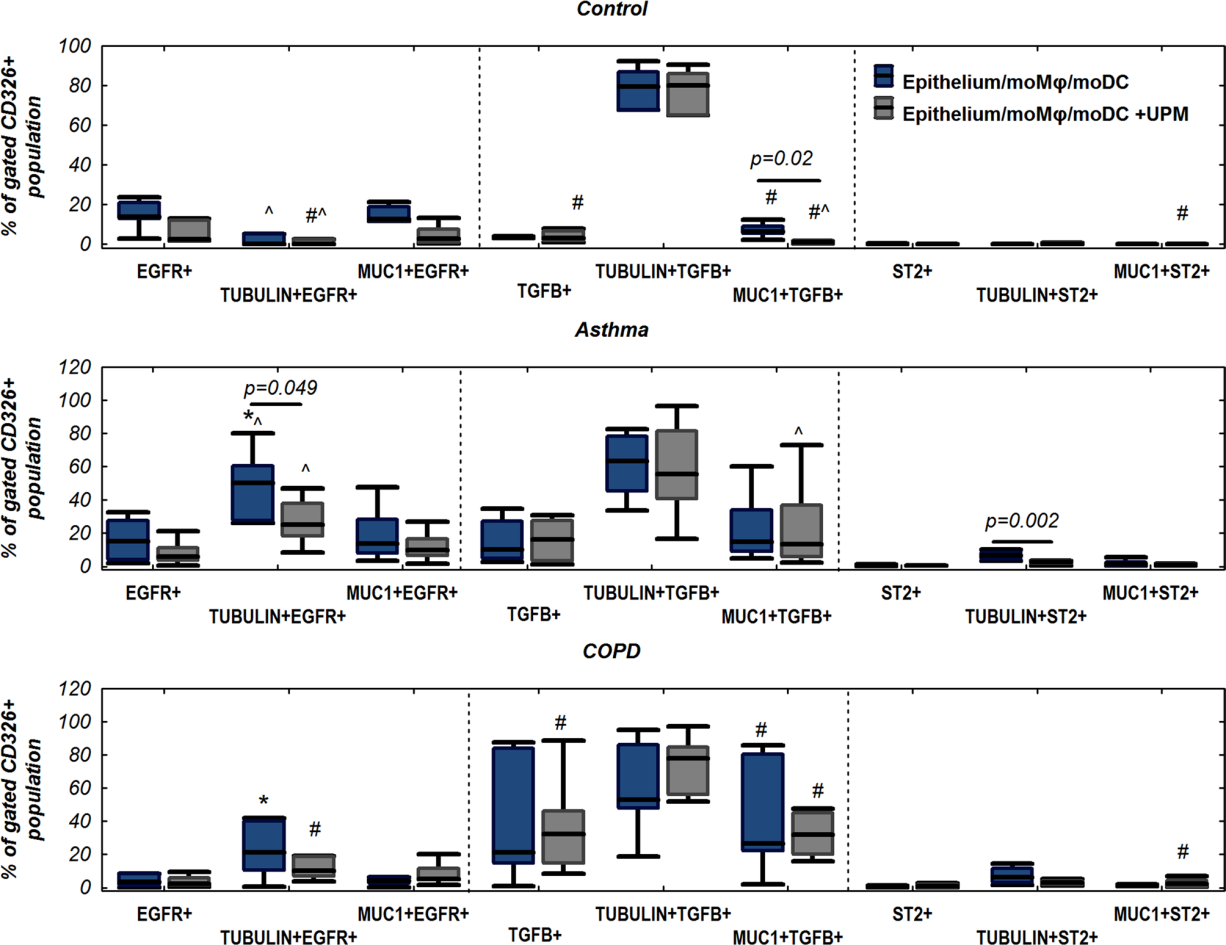
EGFR, TGFβ, and ST2 expression on air–liquid interface (ALI) cultured epithelium upon monocyte derived macrophages (moMφs) and monocyte derived dendritic cells (moDCs), co-cultivation with or without 24 h UPM exposure in control, asthma, or COPD groups. *p* value  < 0.05 calculated using in Mann–Whitney U test: ^ asthma versus control, # COPD versus control, *asthma versus COPD.

The highest proportion of EGFR + epithelial cells was noted in asthma patients. EGFR was expressed mainly in epithelial cells with ciliated phenotype. We did not observe EGFR expression in epithelial cells from controls in unstimulated and UPM stimulated triple co-cultures. UPM exposure decreased the proportion of EGFR + epithelial cells in all evaluated groups with significant changes found in asthma epithelial cells with ciliated phenotype (50.3% (27.7–60.7%) versus 25.2% (18.3–37.9%), *p* = 0.049) (Fig. 13).

TGFβ was expressed in COPD triple co-cultures stimulated with UPM, with its predominance in epithelial cells with ciliated phenotype. The largest proportion of TGFβ + epithelial cells with secretory phenotype was found in unstimulated and UPM stimulated triple co-culture of COPD patients. UPM treatment decreased the proportion of TGFβ + epithelial cells with secretory phenotype in control subjects (6.2% (5.5–9.0%) versus 0.9% (0.5–1.5%), *p* = 0.02) (Fig. 13).

A small proportion of ST2 + epithelial cells was found in all investigated groups. ST2 was expressed most frequently in epithelial cells with ciliated phenotype and was insignificantly higher in patient groups compared to controls. A higher amount of ST2 + epithelial cells with secretory phenotype was observed in triple co-cultures after UPM exposure in COPD compared to the control group. A decreased number of ST2 + epithelial cells with ciliated phenotype was found in asthma after UPM treatment (6.8% (4.4–8.5%) versus 2.8% (1.9–3.3%), *p* = 0.002) (Fig. 13).

## Discussion

Morbidity from asthma and COPD, including acute exacerbations of these diseases, is correlated with an elevated concentration of harmful PM in the atmosphere, suggesting a contribution of air pollution to the pathobiology of obstructive lung diseases. Our study showed a different pattern of epithelial response after UPM exposure in asthma, COPD, and healthy people. Our results revealed that the inflammatory reaction of nasal epithelial cells after UPM treatment was impacted by interactions with moDCs in all studied groups and to a lesser effect with moMφs in COPD. We found different dynamics of changes in the integrity of tight junctions of nasal epithelial cells after 24 h UPM stimulation between asthma, COPD, and control group, and showed, unexpectedly, an increase of the integrity of the cellular barrier of asthmatic epithelium co-cultivated with moMφs only, not related to UPM treatment. The results of our study presented that the inflammatory alternations after UPM exposure are more intense in patients with obstructive lung diseases than in healthy people. Here, for the first time, we characterized the immunological processes induced by airway epithelium after UPM exposure in special regard to interactions with moDCs and moMφs different in asthma, COPD, and healthy people.

The impact of various airborne PM on airway epithelial cells has been well described. Byun et al. analyzed the whole transcriptome of nasal epithelial cells and showed that expression of genes related to inflammation (e.g. IL-8, IL1RL1, PTGS2, ICAM, ADAM8) and adhesion (IL-8, AKAP12, ICAM1, ADAM8, IL-1β) were affected after 24 h UPM exposure^26^. The in vitro studies using ALI cultured airway epithelial cells showed that exposure to PM_2.5_, for example, impacted many processes associated with oxidative stress and pro-inflammatory response in particular: reduced cell viabilities, induced ROS generation, enhanced arginase II levels, heme oxygenase-1 (HO-1), and IL-8 levels^8,27,28^. Our study showed that inflammatory reaction after UPM exposure in the respiratory epithelium is related to cell-to-cell interactions. The inflammation helps the epithelial cells with tolerance and neutralization of noxious stimuli in the airways after air pollution exposure. The crosstalk between immune and epithelial cells regulates Th1- (observed at IL-1β, IL-6 and IL-8 level) inflammatory reaction as it was shown for the control group. IL-6 and IL-8 accelerate the local inflammatory reaction, recruit neutrophils and macrophages for efficient clearance and degradation of inhaled pollutants and apoptotic cells. The role of IL-1β in response to PM seems to be complex. The analysis of whole transcriptome expression in ALI cultured epithelial cells showed the highest changes in expression and function of *IL1A* and *IL1B* among the genes upregulated by PM exposure^29^. It is suggested that IL-1α and IL-1β are key mediators of mucus cell metaplasia in the airways after PM exposure^29,30^. Moreover, increased release of IL-1-β from airway epithelial cells may contribute to abnormal collagen remodeling by airway fibroblasts and be associated with EMT^31^. Taken together, exposure to UPM initiates complex inflammatory processes that are efficient in epithelial cells, which interact with airway immune cells. Prolonged PM exposure may lead to uncontrolled inflammation, acute tissue injury and airway remodeling in healthy people. The results of our study found that the immune responses of epithelial cells in asthma and COPD after PM exposure differ in some aspects of inflammatory pattern from healthy individuals. Moreover, the intensity of inflammation is higher in patients with obstructive lung diseases in our study, especially observed for IL-6 in COPD and IL-8 in both asthma and COPD, and include other mediators e.g., TSLP and IL-33 in asthma. This observation suggests that patients with obstructive lung diseases are more prone to negative effects of air pollution because of epithelial dysfunction and impairment of its protective mechanisms, and more pronounced inflammatory response in the airways.

The epithelial/DCs communication is essential for an immunologic response during allergen or environmental triggers in the lungs. Epithelial cells signal and impact DCs to initiate the allergic immune response by producing cytokines (TSLP, IL-33, IL-25, IL-1α, and GM-CSF)^32^. DCs are activated by components of the epithelial layer: after exposure to epithelium-derived mucin, DCs promoted IL-8-dependent neutrophil migration^33^. Only a few authors evaluated the effect of interactions between epithelial cells and DCs on the inflammatory response after PM stimulation. It has been reported that normal bronchial epithelial (submerged)/DCs co-cultivation enhanced pro-inflammatory responses after PM exposure compared to monocultures. Stimulation of immature DCs to conditioned media from airway epithelial cells exposed to PM_10_ caused DCs maturation, not related to the uric acid pathway^34^. In contrast to DCs, the mucosal PM_10_-facilitated allergic airway response is mediated by uric acid^35^. The results of our work showed that co-cultivation of airway epithelial cells with moDCs upregulated the expression and production of many mediators. In this study, we showed that this is a two-way interaction and DCs also affect epithelium during the response to UPM treatment by upregulation of expression of IL-1β, IL-6, IL-8, or downregulation of MMP7, IL-33 or TSLP. DCs are an additional source of IL-1β and IL-8 during the airway immune response, which accelerates the local inflammation. The results of this work clearly showed that DCs/epithelial interactions significantly affected the inflammatory reaction of epithelial cells after UPM exposure. We suggest that dysfunction of DCs might determine the biological reaction of airway epithelium for air pollution in asthma and COPD patients.

Our study showed that the inflammatory response of epithelial cells after UPM exposure is not mediated by interactions with moMφ, except for IL-1β and IL-6 expression in COPD. These results seem to confirm the earlier observation of Chen et al. who found that pulmonary inflammation in mice after carbon nanoparticle instillation was not stimulated by alveolar macrophages. The macrophages phagocyted the particles but did not interact with epithelial cells as long as macrophages were additionally stimulated with particles^36^. We also found qualitative and quantitative differences in the inflammatory response of respiratory epithelium after UPM stimulation between healthy, asthma, and COPD groups. This reaction is probably related to pathological changes in the epithelium as well as macrophage/DC dysfunction associated with asthma or COPD. The functional abnormality of respiratory epithelium is correlated with the altered expression of various pro-inflammatory cytokines. It is interesting that numerous changes in the mRNA expression for IL-6 or IL-8 were more frequently observed in the control group than in asthma or COPD. This may suggest the impaired function of respiratory epithelium in obstructive lung diseases. The above assumption seems to be supported by the observation that air pollution worsens asthma and COPD symptoms more often than caused pulmonary dysfunction in healthy subjects^37^. The altered immune response in asthma and COPD for air pollution might be also related to disturbed biochemical and molecular signaling between immunological and structural cells. The prolonged exposure to oxidative stress in COPD indicates inherent mitochondrial impairment in macrophages, which resulted in weakened intracellular bacterial killing in respiratory infections related to COPD exacerbations and progression^38^. Our study showed that inflammatory response after UPM exposure is altered with the participation of epithelial/moMφ interactions in COPD only. Together, these facts might suggest a new therapeutic strategy that involves airway epithelium/macrophage biochemical pathways for the effective treatment of air pollution-associated COPD exacerbations.

The results of our work strongly suggest the disruption of the integrity of the epithelial barrier in asthma and as well as in COPD patients. We confirmed the observation of many authors and showed the low integrity of tight junctions of asthmatic but also less frequently studied COPD nasal epithelial cells. Using the epithelial cells from lower airways of patients with different COPD stages, Amatngalim et al. found no differences in barrier function between COPD and non-COPD bronchial epithelial cells^39^. The results of our study suggest a diverse pattern of cell–cell interaction in asthma and COPD compared to controls.

Macrophages are important cells that maintain respiratory epithelial integrity. The airways contain embryonically derived resident macrophages (airspace) and recruited macrophages, which migrate to the respiratory tract in response to inflammatory signal^40^. Macrophages in the airways are motile cells and actively participate in the lung immune response. Macrophages are distributed throughout the lung. Part of them reside in the alveoli (attached or unattached to structural cells), some of them phagocyte and are placed between the epithelial cells (submucosal), others are below the epithelial layer (interstitial) and participate in immune response together with other immune cells or migrate to lymph nodes^41^. Alveolar, submucosal and interstitial macrophages differ in terms of molecular transcriptomic profile^42^. Macrophages are important promoters of tissue repair after e.g. inflammatory lung disease, infection or toxic particles inhalation^43^. Airway macrophages impact epithelial integrity and repair airway injury by efferocytosis of apoptotic cells, interaction with structural cells, and production of growth factors (e.g. VEGF, EGF, fibroblast growth factor, heparin binding growth factor) and cytokines (e.g. GM-CSF, TNF-α, MMPs)^44–48^. In vivo study showed that submucosal macrophages promote epithelial repair and support pseudostratified epithelium growth and regeneration as alternatively activated M2 macrophages^42^. Many authors shoved that phagocytosis and efferocytosis of macrophages in asthma and COPD are impaired. We found that moMφs without UPM treatment produced different cytokines in healthy people and patients with obstructive lung diseases—they do not produce IL-6 in asthma and COPD and secrete more IL-8 in COPD. The results of our study indicated that moMφs of patients with asthma and COPD vary in terms of reaction to UPM exposure from controls suggesting their changed ability for proper protective function in cooperation with epithelial cells after air pollutant response.

Our study showed that the integrity of the cellular barrier was increased in epithelium/moMφs co-cultures compared to epithelium alone of asthma patients; this effect was not UPM-depended. In other in vitro models, the addition of macrophages (THP-1 cell line) did not induce TEER values in pulmonary epithelial cells (Calu-3 cell line) co-cultured with pulmonary endothelial cells (HPMEC-ST1.6R cell line)^49^. In contrast, other authors observed significantly lower TEER values using a similar cell culture model^50^. In similar cell culture configurations as in our study, Lehman et al. found that TEER values of triple-co-cultures were lower than in epithelial monocultures^51^. We believe that our results partially confirm this observation: the addition of moDCs and/or moMφs to epithelial cells decreased TEER values in controls (with the most profound effect in triple co-cultures) but did not change TEER values in asthma and COPD suggesting that this effect is related to their pathology. The use of macrophages in the in vitro system might cause some epithelial morphological changes. The interactions of the moDCs and/or moMφs within the epithelium may elucidate the decrease of the integrity of tight junctions in control di- and triple co-cultures. The normal epithelial cells (in the control group) are probably less tightly connected to each other after moDCs and/or moMφs co-cultivation due to the development of cell–cell interactions in contrast to asthma and COPD where moDCs and/or moMφs co-cultivation with epithelial cells was able to prevent a leakage barrier. It has been shown that moDCs and/or moMφs are able to interact in the triple cell co-culture as a transepithelial network, by building cytoplasmic pseudopodia with epithelium, which might more tightly connect the cells^52^. Our study showed that the interactions between epithelium and macrophages/DCs are different in obstructive lung diseases than in the control group. We can only speculate that this effect is related to epithelial cells which were obtained from more environmental, and disease impacted origin than moDCs and moMφs (from peripheral blood). The hyperreactivity of respiratory epithelial cells in obstructive lung diseases might explain the different effects of UPM treatment on the integrity of the cellular barrier in asthma and COPD compared to controls. The results of our work are partially compatible with other studies. Some of them showed no changes in TEER values after PM exposure with respect to the controls with a simultaneous decrease in the expression of thigh junction proteins^53,54^. Also, the type of in vitro model applied matters: Lehmann et al. found a reduction of TEER in epithelial monocultures after DEP exposure in contrast to triple co-cultures where no changes in TEER value were found^51^. On the other hand, it is known that PM disrupts the airway epithelial barrier via oxidative stress^55,56^. It should be kept in mind that chronic UPM exposure impacts the integrity of the epithelial barrier. The changes in TEER values after 24 h UPM stimulation used in our study reflect the fast changes, whereas a long-term observation could give us the whole picture of the evaluated issue, which is impossible to obtain due to the high toxicity of UPM and complexity of the in vitro model used.

Our study showed that the expression of markers of remodeling was increased in obstructive lung diseases in relation to controls, which might be related to the pathological epithelial dysfunction related to asthma and COPD biology. The components of PM_2.5_ like ROS or PAH are linked to airway remodeling and EMT pathobiology^57,58^. Ambient PM can enhance the expression of ligands for EGFR and phospho‐EGFR^59,60^. PM_2.5_ from wood smoke-induced MUC5AC expression in NCI-H292 cell line via activation of EGFR-extracellular signal-regulated kinase (ERK) signaling^16^. EGFR activation and autocrine production of EGFR ligands are associated with IL-8 expression after DEPs exposure^61^. PM_2.5_ is involved in the increase of epithelial cortical stiffness, which enables mechanical activation of TGFβ^62^. We observed fewer EGFR + and ST2 + ciliated epithelial cells in asthma and TGFβ + secretory epithelial cells in controls after UPM exposure. We can speculate that UPM stimulation causes rapid and huge production of e.g., EGFR ligands (e.g., EGF, transforming growth factor-alpha, betacellulin, and amphiregulin) with a high affinity to the receptor, which prevents binding of fluorochrome-labeled antibodies. Willmarth et al. demonstrated that EGF stimulated EGFR internalization in the normal mammary epithelial cell line MCF10A^63^. In this study, EGF stimulation resulted in significantly less mean fluorescence of EGFR in cells compared to amphiregulin stimulation. The decreased number of EGFR + in flow cytometry in our study might be the result of the receptor internalization by ligands produced by epithelial cells after UPM stimulation and be interpreted as intense receptor activity on triple co-cultured epithelial cells after UPM exposure. We can speculate that air pollution contributes to airway remodeling through EGFR or ST2 on ciliated cells in asthma patients. The reprogrammed and changed function of epithelial cells in the airways might be associated with a more intense reaction of asthmatic patients to air pollution. This observation should be investigated in more detail in the future.

Our study has some limitations. First, our cultures did not contain cells from the lower respiratory tract which are involved in pathobiological processes underlying obstructive lung diseases. This scheme is a simplified model for the evaluation of cell-to-cell interactions, only. Nasal epithelial cells are used as a functional surrogate for bronchial epithelial cells—the main effector structural cells in obstructive lung diseases. On the other hand, the non-invasive obtainment of material led us to create a unique comparison of immunological processes impacted by cell–cell interactions between healthy, asthma and COPD patients, because cells in co-culture configurations came from one patient. Secondly, we did not include healthy smokers as a better comparison for COPD. We believe that a comparison of results from COPD patients with those from non-smoking control subjects, representing a normal airway environment, provided conclusions concerning important processes impacted by UPM in respiratory epithelium. Thirdly, we did not evaluate separate components of UPM. The detailed characteristic of epithelial immunological reactions impacted by PM_10_ or PM_2.5_ in relation to DCs and macrophage interactions with epithelium might be an interesting direction for future research.

## Conclusions

**Figure 14:**
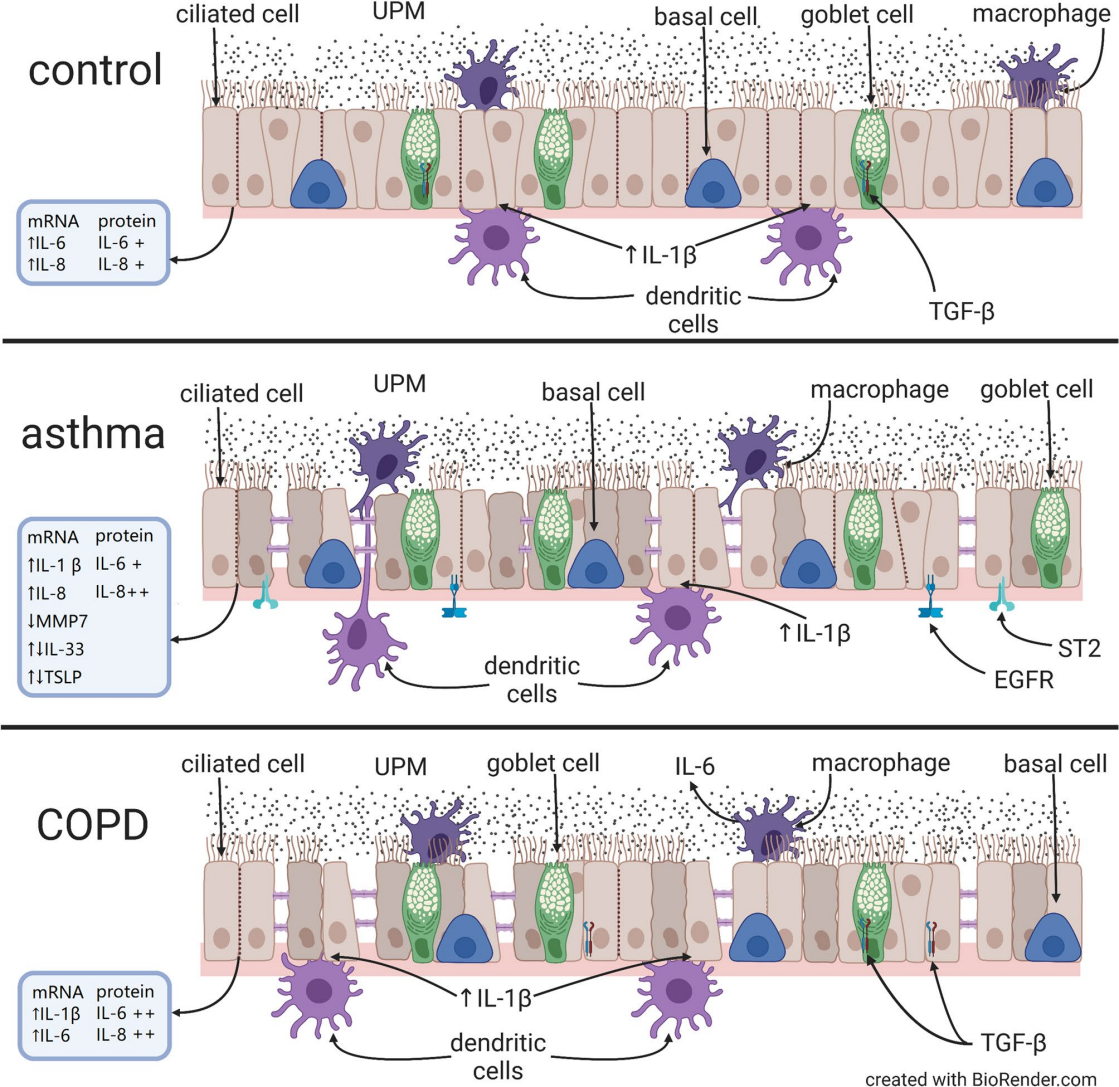
A schematic description of the study results.

The inflammatory response of nasal epithelial cells to UPM stimulation is affected differently by macrophages/epithelial/DCs interactions in healthy people, asthma or COPD patients. We found a strong effect of DCs interactions on the inflammatory reaction of epithelial cells after UPM exposure and speculate that the cross-talk of these cells determinates the local airway response to air pollution. The integrity of epithelial barrier in asthma, and COPD after UPM treatment was affected differently than in controls. The inflammatory alternations after UPM treatment were more intense in patients with obstructive lung diseases than in healthy subjects. We found an increase in the integrity of the cellular barrier of asthmatic epithelium co-cultivated with moMφs not related to UPM exposure. We showed changes in the expression of EGFR + and ST2 + on epithelial cells with ciliated phenotype in asthma, and TGFβ + on epithelial cells with secretory phenotype in controls after UPM exposure. These observations suggest the possible mechanism of airway remodeling through EGFR + and ST2 + associated with air pollution in asthma. A brief summary of the results of this study is illustrated on Fig. 14. The results of our study might help in fully understanding the substantial processes associated with PMs toxicity and its impact on asthma and COPD course.

## Supplementary Information

Supplementary Information.
