# Supplementary material for: Interactions of nasal epithelium with macrophages and dendritic cells variously alter urban PM-induced inflammation in healthy, asthma and COPD
Source: Sci Rep. 2021 Jun 24;11:13259. doi: 10.1038/s41598-021-92626-w (PMC8225888; doi:10.1038/s41598-021-92626-w)
Supplement: Supplementary file 1 — Supplementary Information. [file 41598_2021_92626_MOESM1_ESM.pdf]

Interactions of nasal epithelium with macrophages and dendritic cells variously alter urban PM-induced inflammation in healthy, asthma and COPD

Magdalena Paplinska-Goryca[1&], Paulina Misiukiewicz-Stepien[1,2], Malgorzata Proboszcz[1], Patrycja Nejman-Gryz[1], Katarzyna Gorska[1], Elwira Zajusz-Zubek[3], Rafal Krenke[1]

[1] Department of Internal Medicine, Pulmonary Diseases and Allergy, Medical University of Warsaw, Poland.

[2] Postgraduate School of Molecular Medicine, Medical University of Warsaw, Warsaw, Poland.

[3] Faculty of Energy and Environmental Engineering, Department of Air Protection, Silesian University of Technology, Gliwice, Poland.

Patients' characteristics

This was a prospective, cross-sectional study which involved 8 healthy controls, 10 asthma patients, and 8 patients with COPD. In all patients, the diagnosis of asthma or COPD was previously established according to current Global Initiative for Asthma (GINA) and Global Initiative for Chronic Obstructive Lung Disease (GOLD) recommendations, respectively [14,15]. The following examinations were performed after patient enrolment: medical history, physical examination, spirometry with flow-volume curve, airway obstruction reversibility test (when applicable, performed according to the recommendations of the European Respiratory Society (ERS)), allergy skin prick tests with a panel of fifteen aeroallergens. Disease control was assessed by Asthma Control Test (ACT) in asthmatics and by the COPD Assessment Test (CAT) in patients with COPD. The nasal brushing and peripheral blood sample were obtained from each patient. Exclusion criteria for all asthma and COPD patients were systemic or nasal steroid treatment, disease exacerbation or symptoms of respiratory tract infection in the previous 3 months. The control group consisted of smoking and non-smoking volunteers, without with normal spirometry.

Monocyte derived dendritic cell (moDC) and monocyte derived macrophages (moMφ) culture

Peripheral blood mononuclear cells (PBMCs) were isolated from freshly drawn venous blood by Lymphoprep (Stemcell, Canada) centrifugation. The PBMC monocytes were achieved by adherence in Monocyte Attachment Medium (Promocell, Germany) for 2 hours. Monocyte derived dendritic cells (moDCs) were cultured in X-Vivo 20 medium (Lonza, Austria) supplemented with 40 ng/ml granulocyte-macrophage colony-stimulating factor (GM-CSF) (Stemcell, Canada) for 8 days and 20 ng/ml IL-4 (PromoKine, Germany) for 5 days and 50 ng/ml IL-1β (Stemcell, Canada) and 50 ng/ml TNF-α (Biotechne, R&D Systems, MN, USA) in the 6th day of culture. Monocyte derived macrophages (moMφs) were specialized from PBMC monocytes by stimulation with 20 ng/ml macrophage colony-stimulating factor M-CSF (Stemcell, Canada) for 10 days in Macrophage Base Medium DXF (Promocell, Germany).

The culture of epithelial cells in air liquid interface (ALI)

Epithelial cells were obtained by nasal brushing (Cytobrush Plus GT, CooperSurgical, Germany). The cells were detached from the brush by gentle agitation, centrifuged (300 g, 10 min, room temperature) and treated with Accutase (BD Biosciences, San Jose, CA, USA) for 10 min. in 37°C. The cell pellet was suspended in a total volume of 5 ml of airway epithelial growth medium (Promocell, Germany) containing antibiotics, and seeded into sterile plastic T25 bottles (Thermo Fisher Scientific, MA, USA). Cells were

incubated in a plastic dish for 24 hours at 37°C. The undetached cells were removed and the cell medium changed. The adhered cells were cultured until reached 80% of confluency. $1x10^5$ cells/cm^2 were seeded on the apical surface of 6.5 mm trans well Thin Certs with 0.4 µm pore size and placed in 24 well flat-bottomed plates (Greiner Bio-One, Austria) with epithelial growth medium (Promocell, Germany) in the basal (0.5 ml) and upper (0.2 ml) compartments. After 100% of confluence was reached, cells in the upper chamber were exposed to air, the medium in basal chamber was changed for ALI Maintenance Medium (Stemcell, Canada) and exchanged every 2 day. The ALI epithelium was generated for 21 days.

Triple Co-Culture

Each triple-cell co-culture model will contain three different cell types obtained from one patient. The fully specialized ALI epithelial cells were supplemented with moMφs and moDCs as follows: moDCs were harvested, washed, centrifuged, re-suspended in X-Vivo-20 medium and $2x10^5$ cells in 50 µl medium was added to the basal side of the inserts placed upside down. The dish with the inserts was covered and placed in the incubator for 1.5h–2h. MoMφs were harvested by washing with PBS followed by addition of Cellstripper (Corning, New York, USA) and detached with cell scrapers. A volume of 35 µl Macrophage Medium DXF (Promocell, Germany) containing $1.0x10^5$ cells was added on the apical side of the epithelial monolayer on the insert, forming the upper chamber.

RNA isolation, cDNA synthesis and real time PCR

Total RNA was isolated from the cells using Trizol (Sigma Aldrich, MO, USA). The concentration and purity of isolated RNA was measured on a DU650 spectrophotometer (Beckman Coulter, Brea, CA, USA). Eight microliters of total RNA was used for reverse transcription (Thermo Fisher Scientific, MA, USA). Real-time PCR measurements were performed with an ABI-Prism 7500 Sequence Detector System (Applied Biosystems, Thermo Fisher, MA, USA). For PCR reaction 0.8µl of cDNA was amplified in 16 µl PCR volume, containing a TaqMan master mix (Thermo Fisher Scientific, MA, USA) with 150 nM of specific primers and 100 nM of probe (Table S1 Thermo Fisher Scientific, MA, USA). Each sample was measured in duplicate. The results were expressed as relative quantification units (fold change). Relative quantification values were calculated by the $2^{-\Delta\Delta CT}$ method. 18S rRNA was applied for each sample as an internal control in order to normalize gene expression levels. The mean ΔCT of unstimulated epithelial cells from controls was used as a calibrator for all groups.

Table S1 Sequence of primers used in PCR.

	Forward primer	Reverse primer		Product size
18s rRNA	Hs99999901_s1			187
IL-1β	Hs01555410_m1			91
MMP7	Hs01042796_m1			64
IL-6	CCGGGAACGAAAGAGAAGCT	GCGCTTGTGGAGAAGGAGTT	TCTCCCCTCCAGGAGCCCAGCTA	67
IL-8	GAGCACTCCATAAGGCACAAACT	ATCAGGAAGGCTGCCAAGAG	CCAGGAAGAAACCACCGGAAGGAACC	149
MMP9	GCTCACCTTCACTCGCGTG	CGCGACACCAAACTGGATG	ACAGCCGGGACGCAGACATCG	61
TSLP	CCACTGGTGTTTATAGGGTTCTGA	TCTTGAATTCCCGCTGCAA	TCTCCCCTCCAGGAGCCCAGCTA	79

IL-33	GCCTAGATGAGACACC GAATTAACA	CCAGGGTCAGAAGGG ATGCT	AACTGACTGTCCCTCATG TCCATGGCC	86

Flow cytometry

Human TruStain FcX (Biolegend, San Diego, CA, USA) (5 µl per 100 µl of sample) was added to block non-specific bindings. Cells were stained with antibodies against the surface binding molecules, for epithelial cells: CD326, CD45, EGF, MUC1 (BD Biosciences, San Jose, CA, USA), ST2 (Biotechne, R&D Systems, MN, USA) (Table S2) in BD Horizon Brilliant Stain Buffer (BD Biosciences, San Jose, CA, USA), and incubated for 20 minutes in the dark at room temperature. After washing away the reagents, the cells were fixed and permeabilizated using lysis buffer and permeabilizations solution 2 (BD Biosciences, San Jose, CA, USA), then stained with intracellular marker (TGF-β1, β-tubulin (BD Biosciences, San Jose, CA, USA)) for 20 minutes in the dark.

Cells were analysed by flow cytometry using a FACSCelesta instrument (BD Biosciences, San Jose, CA, USA) equipped with blue (488-nm), violet (405-nm), and red (640-nm) lasers. Unstained cells and compensation beads (BD Biosciences, San Jose, CA, USA) were used to set voltages and create single stain negative and positive controls. Compensation was set to account for spectral overlap between the seven fluorescent channels used in the study. Samples were examined by side scatter area (SSC-A) versus forward scatter area (FSC-A), then using forward scatter height (FSC-H) versus FSC-A to select single cells, eliminating debris and clumped cells from the analysis. At least 50 000 cells in the target gate were collected.

Table S2. The characterisation of fluorochrome-conjugated antibodies for epithelial cells used in flow cytometric analysis

Target	Fluorochrome conjugate	Emission Max (nm)	Clone	Supplier	Catalogue number	Quantity per sample
EGF	BV421	421	EGFR1	BD Biosciences	566254	3 µl
β-TUBULIN	ALEXA FLUOR 488	519	TUJ1	BD Biosciences	560381	7 µl
TGF	PE	578	TW4-9E7	BD Biosciences	562339	3 µl
MUC1	BV605	602	HMFG2	BD Biosciences	747654	3 µl
ST2	APC	660		Biotechne	FAB5231A	7 µl
CD326	BB700	693	EBA-1	BD Biosciences	745841	3 µl
CD45	APC-H7	785	2D1	BD Biosciences	641399	3 µl

Table S3. IL-1β, IL-6, IL-8, MMP7, MMP9, TSLP, IL-33 mRNA expression in air–liquid interface (ALI) cultured nasal epithelium after 24h UPM exposure in multi co-culture schemes in control subjects, asthma and COPD patients.

		Control (n=8)	Asthma (n=10)	COPD (n=8)
	IL-1β mRNA expression (fold change)			
a	Epithelium	-1.1 (-2.3-1.3)	3.7 (2.3-15.0)	2.4 (-1.2-8.3)
b	Epithelium+UPM	-1.4 (-4.3-6.0)	16.1 (5.1-45.8)	5.5 (-0.7-27.9)
c	Epithelium+DC	2.0 (-1.4-38.5)	8.0 (2.4-21.3)	0.1 (-14.1-7.0)
d	Epithelium+DC+UPM	4.5 (-1.5-89.7)	25.9 (13.0-36.2) [f]	3.2 (-1.4-37.6)
e	Epithelium+Mφ	-2.7 (-9.4 – -1.6)	4.1 (-1.7-26.5)	0.2 (-3.0-3.8)
f	Epithelium+Mφ (UPM24h)	-1.6 (-3.2 – -1.1)	3.5 (0.5-9.5) [d]	1.6 (-1.8-3.2)
g	Epithelium+Mφ+UPM	0.0 (-8.3-14.5)	10.2 (-5.6-19.4)	9.3 (1.6-29.7) h
h	Epithelium+Mφ+DC	-2.4 (-6.4 – -1.7)	5.2 (2.2-8.9)	-1.5 (-28.1-2.9) g
i	Epithelium+Mφ+DC+UPM	8.2 (-1.8-46.7)	9.8 (7.4-32.2)	3.4 (-0.2-18.2)
j	Epithelium+Mφ (UPM24h)+DC+UPM	5.6 (-1.6-42.7)	4.3 (1.9-12.0)	1.9 (-0.4-4.4)
	IL-6 mRNA expression (fold change)			
a	Epithelium	-1.3 (-1.7-2.3)	3.7 (-2.1-22.5)	1.4 (-2.1-2.8) [d]
b	Epithelium+UPM	1.1 (-6.5-2.6) d	1.3 (-5.1-16.7)	3.5 (-0.1-8.5) [e,h]
c	Epithelium+DC	-1.4 (-2.7-22.2)	8.1 (-7.2-83.5)	1.6 (-3.6-3.6)
d	Epithelium+DC+UPM	4.0 (3.5-121.1) [b,e,f]	7.7 (-4.5-194.5)	7.0 (2.9-12.2) [a,e,h]
e	Epithelium+Mφ	-3.7 (-16.7– -2.0) [d,i]	1.5 (-9.4-14.9)	-2.4 (-7.0-1.1) [b,d,g]
f	Epithelium+Mφ (UPM24h)	-2.0 (-13.0-2.3) [d]	-0.1 (-19.2-15.9)	2.3 (-4.8-4.0)
g	Epithelium+Mφ+UPM	-5.1 (-30.4-6.4)	1.3 (-1.1-5.2)	9.8 (0.4-29.1) [e,h]
h	Epithelium+Mφ+DC	-2.5 (-33.4-3.0)	4.4 (-1.7-23.2)	-0.3 (-7.6-2.3) [b,d,g]
i	Epithelium+Mφ+DC+UPM	3.6 (-1.2-25.1) [e]	9.0 (-1.3-171.3)	3.8 (1.4-22.3)
j	Epithelium+Mφ (UPM24h)+DC+UPM	1.9 (-1.0-45.9)	1.2 (-3.2-22.6)	-0.3 (-6.1-9.1)
	IL-8 mRNA expression (fold change)			
a	Epithelium	1.2 (-4.9-1.6) [j]	2.7 (-1.9-4.4)	-2.5 (-8.6-0.3)
b	Epithelium+UPM	-2.1 (-4.8 – -1.4) [d,i,j]	1.6 (-3.1-3.8)	-1.8 (-3.5– -0.1)
c	Epithelium+DC	1.5 (-3.0-30.2)	1.6 (-1.2-8.2)	-0.1 (-5.9-1.9)
d	Epithelium+DC+UPM	3.4 (1.7-7.8) [b,e,g]	6.4 (3.4-11.5) [h,j]	-1.3 (-1.8-1.7)
e	Epithelium+Mφ	-2.2 (-3.5-2.0) [d,j]	-1.4 (-3.5-3.3)	-1.7 (-7.0 – -1.1)
f	Epithelium+Mφ (UPM24h)	1.6 (-1.8-2.1)	-1.1 (-1.8-3.4)	-2.1 (-3.1– -0.1)
g	Epithelium+Mφ+UPM	-2.7 (-3.4 – -1.7) [d,j]	2.0 (-4.1-2.5)	-1.4 (-2.5-1.3)
h	Epithelium+Mφ+DC	-2.2 (-5.2-1.5)	1.5 (1.1-3.6) [d]	-1.8 (-8.0-0.1)
i	Epithelium+Mφ+DC+UPM	1.5 (1.3-7.2) [b]	3.5 (1.8-5.7) [j]	-1.5 (-2.4-1.7)

j	Epithelium+Mφ (UPM24h)+DC+UPM	3.0 (1.7-25.4) [a,b,e,g]	-0.1 (-2.8-1.7) [d,i]	-1.8 (-3.1 – -0.1)
	MMP7 mRNA expression (fold change)			
a	Epithelium	-1.8 (-3.1-1.6)	5.6 (3.3-11.5) [j]	4.4 (-4.9-24.2)
b	Epithelium+UPM	-1.6 (-4.6-1.0)	0.1 (-4.2-12.2)	-1.2 (-6.7-9.5)
c	Epithelium+DC	1.6 (-1.8-33.7)	2.2 (-2.6-9.4)	9.4 (-4.7-22.0)
d	Epithelium+DC+UPM	-1.8 (-4.8-8.5)	1.9 (-1.4-9.6)	0.2 (-7.7-6.4)
e	Epithelium+Mφ	-2.4 (-5.3-2.3)	6.1 (1.6-14.3)	11.2 (-2.9-23.2)
f	Epithelium+Mφ (UPM24h)	-3.0 (-3.3-1.8)	2.1 (-5.5-7.4)	0.0 (-7.4-5.5)
g	Epithelium+Mφ+UPM	-2.2 (-4.3 – -1.1)	-1.0 (-2.2-8.5)	2.0 (-5.9-6.5)
h	Epithelium+Mφ+DC	-2.9 (-4.8-1.0)	2.2 (1.5-5.3)	1.2 (-5.0-11.0)
i	Epithelium+Mφ+DC+UPM	-1.4 (-3.3-3.1)	-1.3 (-2.0-12.5)	0.8 (-12.4-7.2)
j	Epithelium+Mφ (UPM24h)+DC+UPM	-2.2 (-3.0-1.1)	1.0 (-6.3-2.6) [a]	-0.7 (-6.3-5.2)
	MMP9 mRNA expression (fold change)			
a	Epithelium	1.2 (-2.0-1.4)	4.9 (2.3-8.8) [c]	-2.0 (-4.7 – -1.4)
b	Epithelium+UPM	-3.4 (-7.3-1.0)	-1.4 (-2.5-2.0)	-3.6 (-6.6 – -1.8)
c	Epithelium+DC	-3.7 (-5.8-6.5)	1.2 (-2.4-1.4) [a]	-4.7 (-6.6 – -2.2)
d	Epithelium+DC+UPM	2.1 (-2.9-5.5)	1.8 (-1.5-3.8)	-3.0 (-14.3-2.3)
e	Epithelium+Mφ	-1.1 (-7.9 – -1.1)	2.7 (1.4-14.7)	-1.5 (-3.3-2.2)
f	Epithelium+Mφ (UPM24h)	-2.0 (-5.3--1.2)	-1.5 (-2.6-5.9)	-2.1 (-7.7--0.2)
g	Epithelium+Mφ+UPM	-2.1 (-2.9-1.1)	-1.1 (-2.6-6.8)	-2.0 (-4.2-2.1)
h	Epithelium+Mφ+DC	-2.8 (-6.1-1.3)	2.2 (-1.6-4.5)	-1.8 (-2.4-0.1)
i	Epithelium+Mφ+DC+UPM	0.0 (-1.5-5.8)	1.6 (-1.2-7.5)	-2.2 (-6.3-0.9)
j	Epithelium+Mφ (UPM24h)+DC+UPM	-1.0 (-4.8-35.0)	2.9 (1.0-6.5)	-3.1 (-23.1-1.3)
	IL-33 mRNA expression (fold change)			
a	Epithelium	-1.0 (-3.0-1.0)	11.6 (2.9-45.5) [j]	6.1 (2.3-35.9)
b	Epithelium+UPM	-2.3 (-5.1– -1.7)	1.3 (-1.2-2.5)	8.3 (1.8-20.5)
c	Epithelium+DC	-1.8 (-5.2-5.4)	2.3 (1.3-4.7) [j]	9.5 (2.3-49.2)
d	Epithelium+DC+UPM	-1.8 (-2.8 – -1.0)	4.2 (1.5-6.0) [j]	4.1 (2.3-15.4)
e	Epithelium+Mφ	-1.4 (-4.1-2.7)	4.4 (-1.1-63.1)	3.9 (2.0-53.7)
f	Epithelium+Mφ (UPM24h)	-1.8 (-3.0-10.2)	2.1 (-0.1-13.2)	9.3 (0.0-44.6)
g	Epithelium+Mφ+UPM	-3.4 (-10.7-4.1)	-1.7 (-4.8-1.6)	5.5 (1.5-18.8)
h	Epithelium+Mφ+DC	-4.5 (-12.1-5.3)	1.3 (-2.4-2.2)	6.4 (3.6-22.8)
i	Epithelium+Mφ+DC+UPM	-2.4 (-2.8-1.4)	1.8 (-1.9-3.1)	3.1 (1.3-27.1)
j	Epithelium+Mφ (UPM24h)+DC+UPM	-2.1 (-2.9-30.0)	-1.1 (-2.8-1.2) [a,c,d]	2.4 (1.4-36.5)
	TSLP mRNA expression (fold change)			
a	Epithelium	1.3 (-4.0-3.0)	7.1 (2.9-22.1) [c,d,i,j]	3.7 (-3.6-10.2)
b	Epithelium+UPM	-5.2 (-12.8 – -2.3) [d]	3.0 (-1.7-6.9)	4.3 (-6.4-14.3)
c	Epithelium+DC	-6.3 (-12.7-11.9)	0.7 (-2.3-4.2) [a]	3.8 (0.9-12.5)
d	Epithelium+DC+UPM	1.4 (1.1-2.9) [b]	1.8 (-1.3-2.4) [a]	8.0 (-0.3-29.7)

e	Epithelium+Mφ	-1.8 (-4.7-2.8)	3.1 (-1.4-5.4)	2.5 (-2.4-13.2)
f	Epithelium+Mφ (UPM24h)	-1.4 (-4.9-1.1)	2.1 (0.3-3.1)	10.1 (-6.0-13.2)
g	Epithelium+Mφ+UPM	-3.3 (-41.5-1.5)	1.7 (-1.6-8.5)	5.4 (-0.1-14.8)
h	Epithelium+Mφ+DC	-8.9 (-34.7-1.5)	2.0 (-2.0-4.5)	2.2 (-2.8-4.6)
i	Epithelium+Mφ+DC+UPM	-2.2 (-2.8-3.1)	-0.1 (-2.0-2.3) **a**	3.6 (-4.3-21.8)
j	Epithelium+Mφ (UPM24h)+DC+UPM	-2.1 (-9.5-355.9)	1.3 (-2.5-2.8) **a**	3.3 (-10.5-21.3)

The results are presented as median and IQR, p-value calculated using Mann–Whitney U test. The p-value<0.05 in comparison to: a - epithelium, b - epithelium+UPM, c - epithelium+moDCs, d – epithelium+moDCs+UPM, e - epithelium+moMφs, f - epithelium+moMφs (24h UPM), g- epithelium +moMφs+UPM, h - epithelium+moMφs+moDCs, i - epithelium+moMφs+moDCs+UPM, j - epithelium +moMφs (24hUMP)+moDCs+UPM

Table S4. IL-1β, IL-6, IL-8 secretion by air–liquid interface (ALI) cultured nasal epithelium after 24h UPM exposure in multi co-culture schemes in control subjects, asthma and COPD patients.

		Control (n=8)	Asthma (n=10)	COPD (n=8)
	IL-1β (pg/ml)			
a	Epithelium	0.8 (0.0-2.3) **c,d,h,i,j**	0.0 (0.0-3.5) **c,d,h,i,j**	0.8 (0.0-2.6) **c,d,f,i,j**
b	Epithelium+UPM	1.7 (0.0-2.3) **c,d,h,i,j**	2.2 (0.9-7.0)**d,e,f,h,i,j**	0.0 (0.0-7.3) **c,d,h,i,j**
c	Epithelium+DC	15.6 (7.1-22.9) **a,b,e,f,g**	6.8 (4.1-8.3) **a,e,f,g**	12.6 (7.4-18.3)**a,b,e,f,g**
d	Epithelium+DC+UPM	12.8 (8.3-18.0) **a,b,e,f,g**	6.8 (6.2-10.4) **a,b,e,f,g**	12.3 (7.5-14.2)**a,b,e,f,g**
e	Epithelium+Mφ	0.0 (0.0-1.8) **c,d,h,i,j**	0.0 (0.0-0.0) **b,c,d,h,i,j**	0.0 (0.0-0.0) **c,d,h,i,j**
f	Epithelium+Mφ (UPM24h)	0.0 (0.0-0.0) **c,d,f,i,j**	0.0 (0.0-0.0) **b,c,d,h,i,j**	0.0 (0.0-0.0) **c,d,h,i,j**
g	Epithelium+Mφ+UPM	0.0 (0.0-0.0) **c,d,h,i,j**	0.8 (0.0-3.2) **c,d,h,i,j**	0.8 (0.0-2.6) **c,d,h,i,j**
h	Epithelium+Mφ+DC	9.2 (5.0-20.9) **a,b,e,f,g**	6.2 (4.1-9.3) **a,b,e,f,g**	11.4 (6.8-18.4) **a,b,e,f,g**
i	Epithelium+Mφ+DC+UPM	11.2 (7.2-20.7) **a,b,e,f,g**	7.6 (5.0-13.5) **a,b,e,f,g**	16.6 (13.4-28.5) **a,b,e,f,g**
j	Epithelium+Mφ (UPM24h)+DC+UPM	12.2 (4.9-17.7) **a,b,e,f,g**	8.3 (6.6-12.0) **a,b,e,f,g**	14.1 (8.0-19.0) **a,b,e,f,g**
	IL-6 (pg/ml)			
a	Epithelium	3.4 (1.1-11.9) **d,i,j**	4.7 (3.5-6.0)	2.7 (1.2-66.5)
b	Epithelium+UPM	11.0 (2.3-88.7)	34.3 (5.8-57.3) **h**	15.8 (9.9-92.7)
c	Epithelium+DC	14.4 (9.5-42.6)	10.5 (1.1-29.0)	15.5 (8.8-63.8)
d	Epithelium+DC+UPM	18.0 (12.7-21.1) **a,e,f**	19.4 (4.8-36.5)	31.3 (11.8-102.1)
e	Epithelium+Mφ	2.2 (0.0-10.5) **d,i,j**	4.0 (1.9-19.6)	9.8 (3.3-20.6) **g,i,j**
f	Epithelium+Mφ (UPM24h)	3.1 (2.1-7.9) **d,i,j**	19.3 (5.1-51.3)	14.6 (9.5-28.0) **g,j**
g	Epithelium+Mφ+UPM	8.9 (0.0-18.0)	25.8 (4.5-68.6)	55.6 (31.9-76.0) **e,f**
h	Epithelium+Mφ+DC	10.2 (4.5-19.1)	5.6 (2.9-11.7) **b,j**	16.1 (9.2-55.8)
i	Epithelium+Mφ+DC+UPM	16.0 (13.3-37.1) **a,e,f**	32.6 (3.9-65.2)	54.5 (19.8-117.4) **e**
j	Epithelium+Mφ (UPM24h)+DC+UPM	15.2 (12.3-19.1) **a,e,f**	34.6 (6.0-54.3) **h**	54.4 (18.4-85.5) **e,f**
	IL-8 (pg/ml)			

a	Epithelium	168.6 (143.2-196.5)	184.9 (115.9-321.9) [d,g,j]	311.3 (165.5-1330.6)
b	Epithelium+UPM	180.3 (171.3-184.6)	358.4 (262.5-566.4)	417.8 (255.0-1009.7)
c	Epithelium+DC	249.9 (194.2-264.0) [g]	276.4 (187.0-533.6) [d,j]	461.5 (410.0-1017.3)
d	Epithelium+DC+UPM	227.8 (219.6-297.5) [f,g]	607.7 (442.9-804.5) [a,c,e,h]	431.0 (320.8-544.5)
e	Epithelium+Mφ	199.8 (132.4-232.8)	288.4 (182.3-405.6) [d,j]	403.5 (237.3-549.7) [j]
f	Epithelium+Mφ (UPM24h)	105.2 (84.9-199.0) [d,i]	310.4 (199.9-506.2) [j]	368.5 (247.6-1121.7)
g	Epithelium+Mφ+UPM	163.1 (110.5-194.0) [c,d,i]	407.1 (308.8-507.8) [a]	423.1 (308.3-1643.6)
h	Epithelium+Mφ+DC	203.8 (148.9-244.9)	315.9 (168.3-500.1) [d,j]	482.1 (350.0-1161.0)
i	Epithelium+Mφ+DC+UPM	238.2 (229.6-247.9) [f,g]	363.7 (283.9-592.4)	593.2 (455.0-1539.2)
j	Epithelium+Mφ (UPM24h)+DC+UPM	226.2 (152.0-289.5)	614.4 (425.4-764.8) [a,c,e,f,h]	708.1 (533.6-921.5) [e]

The results are presented as median and IQR, p-value calculated using Mann–Whitney U test. The p-value<0.05 in comparison to: a - epithelium, b - epithelium+UPM, c - epithelium+moDCs, d – epithelium+moDCs+UPM, e - epithelium+moMφs, f - epithelium+moMφs (24h UPM), g- epithelium +moMφs+UPM, h - epithelium+moMφs+moDCs, i - epithelium+moMφs+moDCs+UPM, j - epithelium +moMφs (24hUMP)+moDCs+UPM

Table S5 The comparison (Mann-Whitney test) of IL-1β protein concentration with or without UPM stimulation in nasal epithelial cells cultured in air liquid interference (ALI) alone or co-cultured with monocyte derived macrophages (moMφs) and/or monocyte derived dendritic cells (moDCs) in control group

control	Epithelium	Epithelium +UPM	Epithelium +DC	Epithelium +DC +UPM	Epithelium +Mφ	Epithelium +Mφ(24h UPM)	Epithelium +Mφ +UPM	Epithelium +Mφ +DC	Epithelium +Mφ +DC +UPM	Epithelium +Mφ (24hUMP) +DC +UPM
Epithelium		0.837	0.001	0.0002	0.299	0.227	0.335	0.0003	0.001	0.00003
Epithelium +UPM	0.837		0.022	0.017	0.259	0.209	0.295	0.014	0.035	0.007
Epithelium +DC	0.0008	0.022		0.505	0.001	0.001	0.002	0.573	0.699	0.553
Epithelium +DC+UPM	0.0002	0.017	0.505		0.001	0.001	0.001	0.694	0.836	0.711
Epithelium +Mφ	0.299	0.259	0.001	0.001		0.805	0.836	0.0003	0.001	0.00004
Epithelium +Mφ (24h UPM)	0.227	0.209	0.001	0.001	0.805		0.945	0.0003	0.001	0.00004
Epithelium +Mφ+UPM	0.335	0.295	0.002	0.001	0.836	0.945		0.001	0.002	0.0001
Epithelium +Mφ+DC	0.0003	0.014	0.573	0.694	0.0003	0.000	0.001		0.950	0.970
Epithelium +Mφ+DC +UPM	0.001	0.035	0.699	0.836	0.001	0.001	0.002	0.950		0.750
Epithelium+Mφ (24hUMP)+DC+ UPM	0.00003	0.007	0.553	0.711	0.00004	0.00004	0.0001	0.970	0.750	

Results of pairwise comparisons between groups. P-values from Mann-Whitney test are given above the diagonal. Proportion of means (group given in a column name divided by group given in a row name) are given below the diagonal.

Table S6 The comparison (Mann-Whitney test) of IL-1β protein concentration with or without UPM stimulation in nasal epithelial cells cultured in air liquid interference (ALI) alone or co-cultured with monocyte derived macrophages (moMφs) and/or monocyte derived dendritic cells (moDCs) in asthma group

asthma	Epithelium	Epithelium +UPM	Epithelium +DC	Epithelium +DC +UPM	Epithelium +Mφ	Epithelium +Mφ(24h UPM)	Epithelium +Mφ +UPM	Epithelium +Mφ +DC	Epithelium +Mφ +DC +UPM	Epithelium +Mφ (24hUMP) +DC +UPM
Epithelium		0.382	0.003	0.0003	0.328	0.328	0.798	0.001	0.0003	0.00002
Epithelium +UPM	0.382		0.130	0.038	0.038	0.021	0.328	0.045	0.021	0.016
Epithelium +DC	0.003	0.130		0.505	0.0003	0.0003	0.005	1.000	0.505	0.238
Epithelium +DC+UPM	0.0003	0.038	0.505		0.0002	0.0002	0.0003	0.417	0.959	0.528
Epithelium +Mφ	0.328	0.038	0.0003	0.0002		0.959	0.234	0.00001	0.0002	0.00001
Epithelium +Mφ (24h UPM)	0.328	0.021	0.0003	0.0002	0.959		0.195	0.000003	0.0002	0.00001
Epithelium +Mφ+UPM	0.798	0.328	0.005	0.0003	0.234	0.195		0.001	0.002	0.0001
Epithelium +Mφ+DC	0.001	0.045	1.000	0.417	0.00001	0.000003	0.001		0.417	0.184
Epithelium +Mφ+DC +UPM	0.0003	0.021	0.505	0.959	0.0002	0.0002	0.002	0.417		0.928
Epithelium+Mφ (24hUMP)+DC+ UPM	0.00002	0.016	0.238	0.528	0.00001	0.00001	0.0001	0.184	0.928	

Results of pairwise comparisons between groups. P-values from Mann-Whitney test are given above the diagonal. Proportion of means (group given in a column name divided by group given in a row name) are given below the diagonal.

Table S7 The comparison (Mann-Whitney test) of IL-1β protein concentration with or without UPM stimulation in nasal epithelial cells cultured in air liquid interference (ALI) alone or co-cultured with monocyte derived macrophages (moMφs) and/or monocyte derived dendritic cells (moDCs) in COPD group

COPD	Epithelium	Epithelium +UPM	Epithelium +DC	Epithelium +DC +UPM	Epithelium +Mφ	Epithelium +Mφ(24h UPM)	Epithelium +Mφ +UPM	Epithelium +Mφ +DC	Epithelium +Mφ +DC +UPM	Epithelium +Mφ (24hUMP) +DC +UPM
Epithelium		0.721	0.002	0.002	0.328	0.328	1.000	0.002	0.001	0.0001
Epithelium +UPM	0.721		0.038	0.050	0.645	0.645	0.721	0.023	0.010	0.011
Epithelium +DC	0.002	0.038		0.721	0.001	0.003	0.001	0.834	0.442	0.697
Epithelium +DC+UPM	0.002	0.050	0.721		0.001	0.005	0.002	0.928	0.105	0.490
Epithelium +Mφ	0.328	0.645	0.001	0.001		0.959	0.328	0.001	0.0003	0.0001
Epithelium +Mφ (24h UPM)	0.328	0.645	0.003	0.005	0.959		0.328	0.001	0.001	0.0002
Epithelium +Mφ+UPM	1.000	0.721	0.001	0.002	0.328	0.328		0.001	0.0003	0.00003
Epithelium +Mφ+DC	0.002	0.023	0.834	0.928	0.001	0.001	0.001		0.264	0.515
Epithelium +Mφ+DC +UPM	0.001	0.010	0.442	0.105	0.0003	0.001	0.0003	0.264		0.417
Epithelium+Mφ (24hUMP)+DC+ UPM	0.0001	0.011	0.697	0.490	0.0001	0.0002	0.00003	0.515	0.417	

Results of pairwise comparisons between groups. P-values from Mann-Whitney test are given above the diagonal. Proportion of means (group given in a column name divided by group given in a row name) are given below the diagonal.

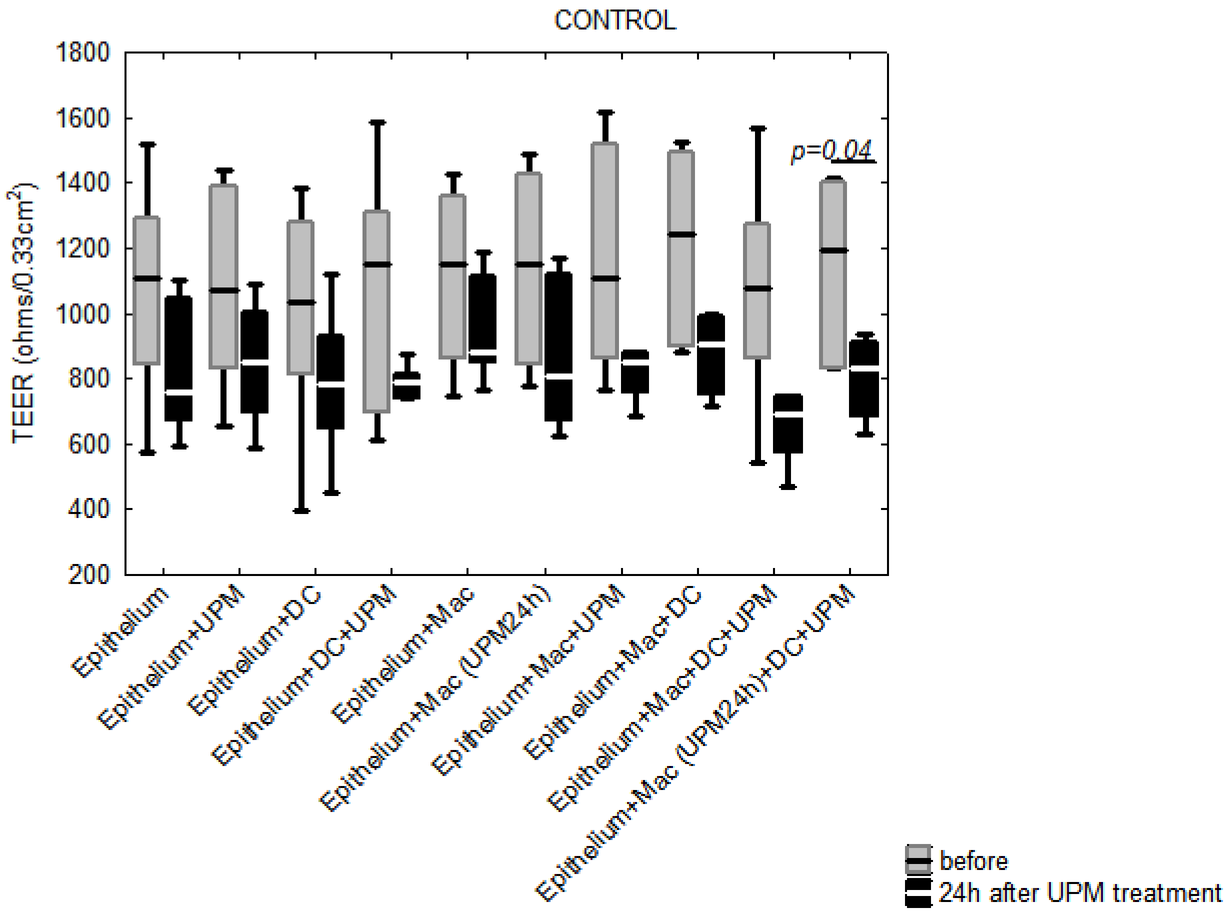

Figure S1 Transepithelial electrical resistance (TEER) after 24h of nasal epithelial cells from healthy donors cultured in air–liquid interface (ALI) conditions in multi co-culture models and stimulated with UPM.

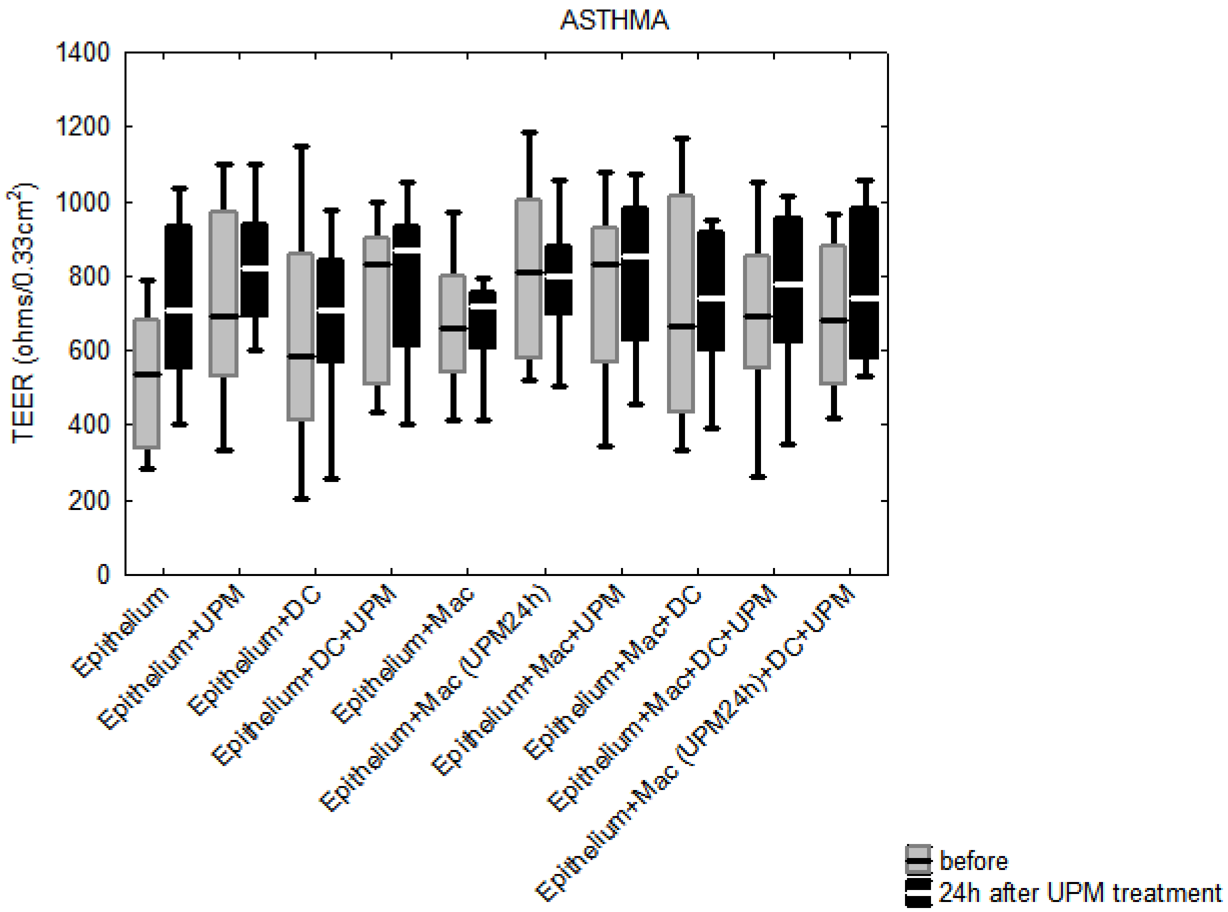

Figure S2. Transepithelial electrical resistance (TEER) after 24h of nasal epithelial cells from asthma patients cultured in air–liquid interface (ALI) conditions in multi co-culture models and stimulated with UPM.

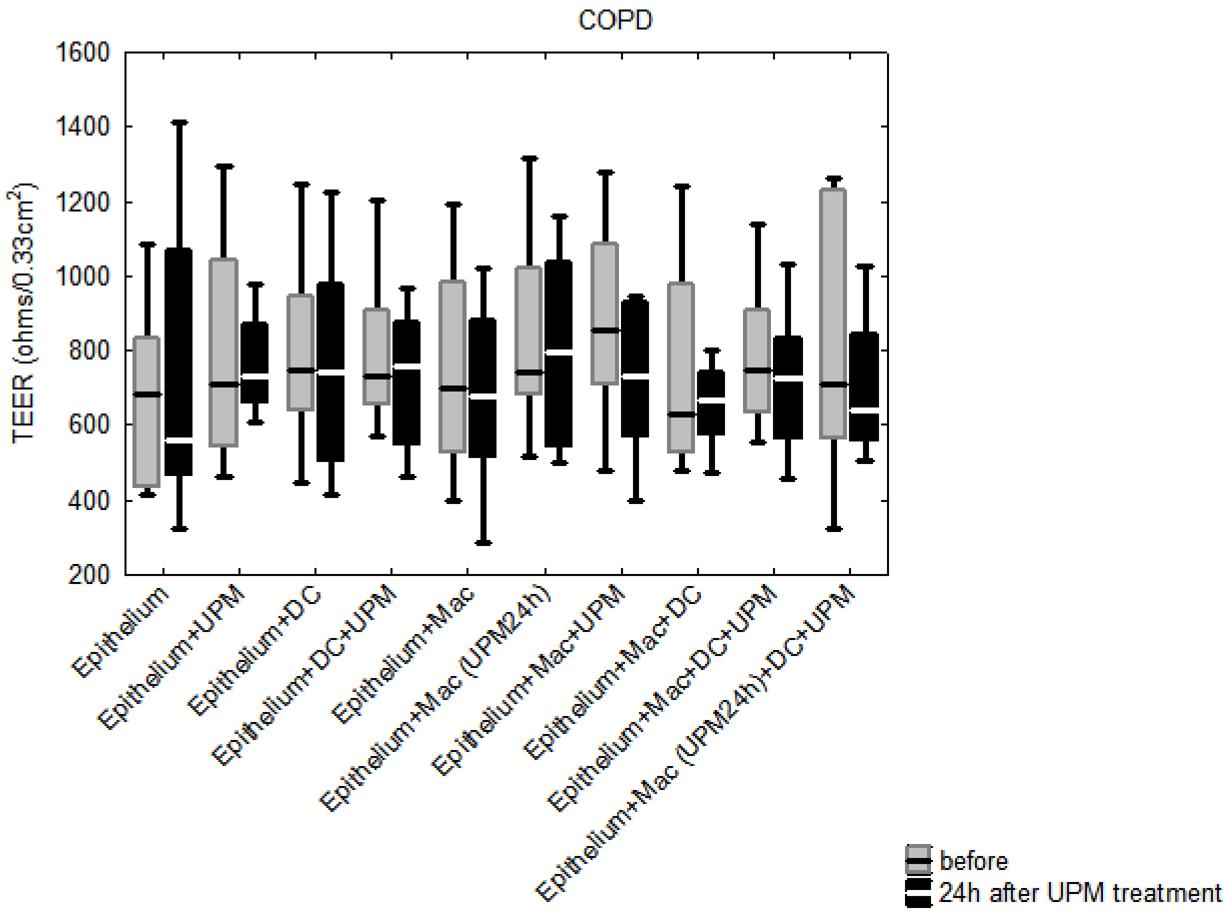

Figure S3. Transepithelial electrical resistance (TEER) after 24h of nasal epithelial cells from COPD patients cultured in air–liquid interface (ALI) conditions in multi co-culture models and stimulated with UPM.
